# Close proximity interactions support transmission of ESBL-*K*. *pneumoniae* but not ESBL-*E*. *coli* in healthcare settings

**DOI:** 10.1371/journal.pcbi.1006496

**Published:** 2019-05-30

**Authors:** Audrey Duval, Thomas Obadia, Pierre-Yves Boëlle, Eric Fleury, Jean-Louis Herrmann, Didier Guillemot, Laura Temime, Lulla Opatowski, the i-Bird Study group

**Affiliations:** 1 Equipe PheMI, unité B2PHI, Inserm, Université de Versailles Saint Quentin, Institut Pasteur,Paris, France; 2 Malaria: Parasites & Hosts Unit, Department of Parasites & Insect Vectors, Institut Pasteur,Paris, France; 3 Institut Pasteur—Bioinformatics and Biostatistics Hub—C3BI, USR 3756 IP CNRS—Paris, France; 4 Sorbonne Université, INSERM, Institut Pierre Louis d'Epidémiologie et de Santé Publique, PARIS France; 5 Univ Lyon, Cnrs, ENS de Lyon, Inria, UCB Lyon 1, LIP UMR 5668, Lyon, FRANCE; 6 INSERM U1173, UFR Simone Veil, Versailles-Saint-Quentin University, Saint-Quentin en Yvelines, France AP-HP, Service de Microbiologie, Hôpital Raymond Poincaré, Garches, France; 7 Laboratoire MESuRS, Conservatoire national des Arts et Métiers, Paris, France; 8 Institut Pasteur, Cnam, unité PACRI, Paris, France; University of California, Los Angeles, UNITED STATES

## Abstract

Antibiotic-resistance of hospital-acquired infections is a major public health issue. The worldwide emergence and diffusion of extended-spectrum β-lactamase (ESBL)-producing Enterobacteriaceae, including *Escherichia coli* (ESBL-EC) and *Klebsiella pneumoniae* (ESBL-KP), is of particular concern. Preventing their nosocomial spread requires understanding their transmission. Using Close Proximity Interactions (CPIs), measured by wearable sensors, and weekly ESBL-EC–and ESBL-KP–carriage data, we traced their possible transmission paths among 329 patients in a 200-bed long-term care facility over 4 months. Based on phenotypically defined resistance profiles to 12 antibiotics only, new bacterial acquisitions were tracked. Extending a previously proposed statistical method, the CPI network’s ability to support observed incident-colonization episodes of ESBL-EC and ESBL-KP was tested. Finally, mathematical modeling based on our findings assessed the effect of several infection-control measures. A potential infector was identified in the CPI network for 80% (16/20) of ESBL-KP acquisition episodes. The lengths of CPI paths between ESBL-KP incident cases and their potential infectors were shorter than predicted by chance (P = 0.02), indicating that CPI-network relationships were consistent with dissemination. Potential ESBL-EC infectors were identified for 54% (19/35) of the acquisitions, with longer-than-expected lengths of CPI paths. These contrasting results yielded differing impacts of infection control scenarios, with contact reduction interventions proving less effective for ESBL-EC than for ESBL-KP. These results highlight the widely variable transmission patterns among ESBL-producing Enterobacteriaceae species. CPI networks supported ESBL-KP, but not ESBL-EC spread. These outcomes could help design more specific surveillance and control strategies to prevent in-hospital Enterobacteriaceae dissemination.

## Author summary

Tracing extended-spectrum β-lactamase (ESBL) dissemination in hospitals is an important step in the fight against the spread of multi-drug resistant bacteria. Indeed, understanding ESBL spreading dynamics will help identify efficient control interventions. In the i-Bird study, patients and hospital staff from a French long-term care facility carried a wearable sensor to capture their interactions at less than 1.5 meters, every 30 seconds over a 4-month period. Every week, patients were also swabbed to detect carriage of ESBL-producing Enterobacteriaceae. Based on the analysis of these longitudinal data, this study shows that ESBL-producing *Klebsiella pneumoniae* (ESBL-KP) mostly spreads during close-proximity interactions between individuals, while this is not the case for ESBL-producing *Escherichia coli* (ESBL-EC), suggesting that ESBL-KP but not ESBL-EC may be controlled by contact reduction interventions.

## Introduction

Multidrug resistant (MDR)-Enterobacteriaceae are a common cause of healthcare-associated and community-acquired infections in humans [1], due to the increase over recent years of third-generation cephalosporin, fluoroquinolone and carbapenem resistances [2,3], leading to difficulties finding appropriate treatment and increased mortality and morbidity. The recent emergence of colistin resistance among Gram-negative bacteria also raises new concerns [4]. According to a recent World Health Organization (WHO) assessment, one of the greatest threats to human health is posed by extended-spectrum β-lactamase (ESBL)-producing *Escherichia coli* (ESBL-EC) and ESBL-producing *Klebsiella pneumoniae* (ESBL-KP), which are listed among the priority 1 pathogens for research and development of new antibiotics [5]. These bacteria mostly cause bloodstream, urinary tract and respiratory infections [3].

The infections burden of those bacteria is predominantly in hospitals worldwide. In a WHO review, *E*. *coli* (20.1%) was the most frequent single pathogen causing healthcare-associated infections in mixed patient populations [6]. A large US prevalence survey found *E*. *coli* and *K*. *pneumoniae* to be responsible for 20% of all healthcare-associated infections and 50% of healthcare-associated urinary tract infections [7]. In a recent pan-European cohort, bacteremia caused by ESBL-producing Enterobacteriaceae increased mortality (hazard ratio (HR): 1.63; 95% confidence interval (CI): 1.13–2.35), lengths of stay (4.9; 95% CI: 1.1–8.7 days), and healthcare-associated costs compared with non-ESBL-producing strains [8]. However, to control the threat of these bacteria in hospital settings, more insight is needed regarding their transmission routes [9].

New technologies to measure close proximity interactions (CPIs) by wireless sensors [10,11] have been implemented in hospital investigations [12–16]. CPIs are assumed to be a proxy of human contacts that support human-to-human microorganisms transmission [17–20]. In an earlier study, CPI networks were shown to be a significant support of *Staphylococcus aureus* transmission [21,22].

In this study, we exploited the original longitudinal observational i-Bird (Individual-Based Investigation of Resistance Dissemination) data collected in a 200-bed long-term care facility (LTCF). CPIs between patients and hospital staff were recorded every 30 s over a 4-month period and rectal swabs were collected weekly from patients to test for Enterobacteriaceae carriage. We separately examined the role of CPIs in ESBL-EC and ESBL-KP spread [9]. Using a mathematical model, we tested whether CPI information could be useful in designing and organizing control interventions in LTCFs.

## Results

### ESBL-EC and ESBL-KP colonization

The i-Bird study included 329 patients. The weekly average carriage prevalence of ESBL-producing Enterobacteriaceae was 16.8%. The predominant species were *E*. *coli* and *K*. *pneumoniae*, with on average 11.5% of patients colonized weekly by an ESBL-EC, and 3.7% by an ESBL-KP (Table 1). Over the 4 months of the study, 203 patients were admitted and swabbed at admission (S1 Fig); 16 of those patients carried an ESBL-EC and 2 an ESBL-KP on admission, representing respective importation rates of 8% and 1%. Overall, 35 incident-colonization episodes were observed for ESBL-EC (acquisition rate: 0.66%/week), and 20 for ESBL-KP (acquisition rate: 0.38%/week).

**Table 1:** Characteristics of the extended-spectrum β-lactamase ESBL-producing *E*. *coli* (ESBL-EC)- and *K*. *pneumoniae* (ESBL-KP)-carrier population. Details about colonized patients, ward prevalence, incidence and CPIs description of colonized patients are summarized below.

Characteristic	Ward 1	Ward 2	Ward 3	Ward 4	Ward 5	LTCF
No. of patients per week (median)	36	27	21	28	16	128
ESBL-EC						
Age (median (range))	53.23 (31–70)	54.2 (40–70)	57.5 (32–80)	48.25 (27–80)	84.36 (76–100)	60.82 (27–100)
Gender (% female)	53.85	40	62.5	50	63.64	33.33
Total number of colonized patients by at least one ESBL-EC	13	5	8	8	11	45
Average weekly prevalence (%)	12.79	8.38	8.41	7.66	14.85	11.51
Average incidence (acquisitions/100 patients/week)	2.71	1.16	1.35	1.45	4.23	1.96
Mean no. of daily distinct CPIs (SD)	16.33 (10.1)	9.97 (2)	12.71 (4)	13.7 (4)	7.87 (2.3)	12.44 (6.7)
With patients	8.11 (6.7)	3.52 (1.5)	6.56 (2.6)	7.42 (3.9)	3.11 (0.8)	5.98 (4.5)
With hospital staff	8.22 (3.7)	6.45 (1.2)	6.16 (2)	6.29 (2.4)	4.76 (1.8)	6.47 (2.8)
Mean daily cumulative duration of CPI (SD)	43.83 (13.9)	32.42 (18.2)	28.15 (26.7)	27.62 (14.8)	54.36 (26.7)	39.47 (22.5)
With patients	89.54 (44.4)	57.11 (40.3)	43.47 (36.4)	46.93 (42.4)	109.54 (49)	75.06 (49.5)
With hospital staff	8.05 (2.4)	15.62 (13.7)	15.71 (19.6)	13.68 (9)	15.24 (25)	13.01 (15.7)
ESBL-KP						
Age (median (range))	53 (34–70)	40	53.5 (27–70)	53 (44–62)	0	52.39 (27–70)
Gender (% female)	10	100	75	100	0	6.67
Total number of colonized patients by at least one ESBL-KP	10	1	4	3	0	18
Average weekly prevalence (%)	14.4	0.26	1.61	0.73	0	3.73
Average incidence (acquisitions/100 patients/week)	4.16	0	1.13	0.3	0	1.15
Mean no. of daily distinct CPIs (SD)	13.61 (1.2)	12	13.56 (5.8)	8.17 (0.8)	0	12.6 (3.3)
With patients	6.48 (1.4)	5.96	7.99 (4.4)	4.26 (0.4)	0	6.42 (2.4)
With hospital staff	7.13 (1.2)	6.04	5.57 (3)	3.9 (1.1)	0	6.19 (2)
Mean daily cumulative duration (SD)	52.33 (18.6)	7.7	25.01 (26.3)	33.22 (7.9)	0	40.6 (22.9)
With patients	105.52 (50.2)	10.75	36.12 (36.6)	47.21 (7.9)	0	75.12 (53.5)
With hospital staff	10.64 (3.4)	3.85	13.34 (19.8)	16.65 (8.2)	0	11.86 (9.6)

Prevalence and incidence of ESBL-EC were the highest in the geriatric ward 5, with a weekly average of 15% of colonized patients, and an incidence of 4% per week; whereas ESBL-KP prevalence and incidence were the highest in the neurology ward 1 (14% and 4% per week, Table 1). Colonized ward 1 patients had the highest average daily distinct CPIs (16.33 ± 10.1 CPIs per day and 13.61 ± 1.2 CPIs per day, for ESBL-EC and ESBL-KP respectively), equally distributed in patients and hospital staff, while the highest cumulative CPI duration was found for ESBL-EC–colonized ward 5 patients.

### Are ESBL-EC and ESBL-KP transmissions supported by CPIs?

Two ESBL-producing Enterobacteriaceae isolates were assumed to be similar when they belonged to the same species (EC or KP) and had the same resistance-status pattern to 12 selected antibiotics, allowing for susceptible to intermediate (S–I) or intermediate to resistance (I–R) differences. Thirty-five incident-colonization episodes (in which a patient was found to be colonized during a given week by an isolate she/he was not carrying the preceding week) were identified for ESBL-EC and 20 for ESBL-KP. For each incident-colonization episode, “transmission candidates”, i.e. patients who carried the most similar isolate as the case over the preceding 4 weeks, were identified; among transmission candidates, those who were linked to the case via the shortest distance (defined as the number of edges between the two, i.e. length of CPI path) on the CPI-network were called “potential infectors”. For both species, incident-colonization episodes were mostly resolved during the preceding week: a potential infector had been identified during the first week preceding the episode for 56% and 63% of all ESBL-KP and ESBL-EC episodes respectively.

To determine whether CPIs could explain transmission, we tested whether observed distances along the CPI-network between a case and their closest potential infectors were comparable to distances expected under the null hypothesis of independence between CPIs and carriage data. Expected distances were computed as the average of distances obtained from 200 simulations using randomly permutated carriage data.

#### Transmission of ESBL-EC

**Fig 1:**
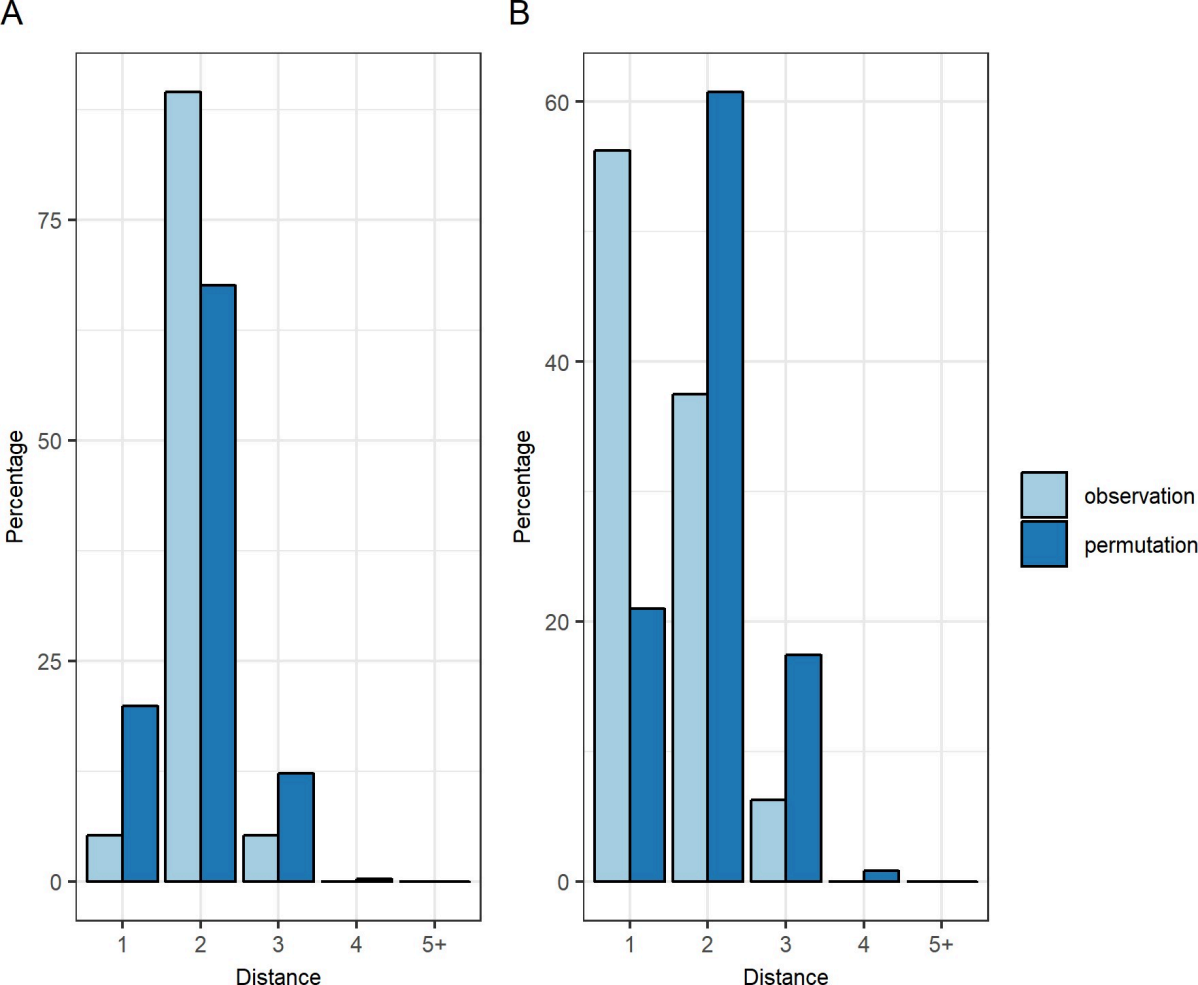
Distribution of distances between acquisition cases and their closest potential infector. Comparison between observed data (light blue) and random permutated data (dark blue). For each incident-colonisation case, potential infectors were selected as the closest in the CPI-network of all candidates sharing the most similar isolate as the case in the preceding 4 weeks. (A) ESBL-EC distribution. (B) ESBL-KP distribution. Here distance is the number of edges between two individuals in the network.

No carrier of the most similar isolate was found over the preceding 4 weeks (transmission candidate) for 13 of the 35 incident-colonization episodes. For 3 additional episodes, no potential infector was found throughout the network, resulting in a total of 16/35 unresolved episodes. Observed and expected case-to-potential infector distances did not differ significantly based on the remaining 19 resolved episodes (P = 0.25, Wilcoxon signed rank paired test). Indeed, more direct CPIs (i.e., distance-1) between cases and their closest potential infectors were found in the permutated data than in the observed data (Fig 1A, 20% and 5% respectively). Conversely, more distance-2 were found in the observed data.

#### Transmission of ESBL-KP

Only 4 of the 20 episodes were not resolved for ESBL-KP i.e. no potential infectors were found. The case-to-potential infector distances differed significantly between observed and simulated datasets for the 16 resolved episodes. That distance was shorter than expected by chance (P = 0.025, Wilcoxon signed rank paired test), suggesting that ESBL-KP transmission was indeed supported by CPIs. There were also more direct CPIs (distance-1) between incident-colonization episodes and their closest potential infector than expected by chance (Fig 1B, 56% vs. 21%).

#### Intermediaries

When looking more precisely at distance-2 between incident cases and their closest potential infector, and more particularly at the distribution of status of intermediaries (i.e. patients or hospital staff), observed and permutated data differed clearly for both species. More patient intermediaries in the observed data for ESBL-KP and more hospital staff for ESBL-EC (S2 Fig) were found.

### Sensitivity analyses

#### Sensitivity of the results to the most similar isolate definition

As expected, a stricter definition of similarity between isolates, taking into account S–I or I–R mismatches in addition to S–R, led to identifying more incident-colonization episodes (49 vs. 35 for ESBL-EC and 49 vs. 20 for ESBL-KP, Table 2). With this scenario, a lower percentage of episodes was resolved (41% vs. 54% for ESBL-EC and 33% vs. 80% for ESBL-KP), especially for ESBL-KP but in absolute values, almost similar numbers of episodes were resolved (20/49 vs. 19/35 for ESBL-EC and 16/49 vs. 16/20 for ESBL-KP). Pertinently, application of the strict definition did not change previous conclusions. Case-to-potential infector distances differed significantly between the observed and permutated data for ESBL-KP, infectors were found more frequently in direct contact (ratio of 3.1, P = 0.009), and no significant difference was seen for ESBL-EC (P = 0.29).

**Table 2:** Sensitivity analyses of transmission definition according to the stricter or baseline definition*.

Definition	ESBL-EC	ESBL-KP
Stricter: complete 12-antibiotic sequence		
No. of incident-colonization episodes	49	49
Total resolved episodes †, %	41	33
Resolved episodes the preceding week ‡, %	60	50
Ratio of distance-1 (observed/expected)	0.538	3.149
*P* §	0.287	0.009
Baseline: 12-antibiotic sequence allowing for S-I and I-R differences		
No. of incident-colonization episodes	35	20
Total resolved episodes †, %	54	80
Resolved episodes the preceding week ‡, %	63	56
Ratio of distance-1 (observed/expected)	0.264	2.676
*P*§	0.243	0.025

*Stricter considered two bacteria identical when they were the same species and had the same 12-antibiotic resistance profile; baseline allowed susceptible–intermediate and/or intermediate–resistance differences.

†A potential infector was found.

‡The number of incident-colonization episodes with a potential infector found during the previous week divided by the total number of incident-colonization episodes with a potential infector found over the previous 4 weeks.

§Wilcoxon signed-rank paired test comparing observed vs. permutated distance CPIs.

Extended-spectrum β-lactamase-producing *E*. *coli*, ESBL-EC; extended-spectrum β-lactamase-producing *K*. *pneumoniae*, ESBL-KP; CPI, close-proximity interaction.

#### Sensitivity of the results to the period of investigation

Varying the duration of the investigation period for transmission candidates from 2 up to the entire 17-week participation period did not affect the results: more distance-1 than expected by chance were always found for ESBL-KP and never for ESBL-EC (Table 3). The percentages of resolved episodes increased with the investigation-period duration. For instance, for ESBL-KP, 65% of episodes were resolved for the investigation periods was 2–3 weeks, but this reached 90% for 8 and 17 weeks.

**Table 3:** Sensitivity analyses of 2-, 3-, 8- or 17-week windows of investigation compared to baseline for transmission candidates.

Preceding periods	ESBL-EC	ESBL-KP
2 weeks		
Total episodes resolved*, %	43	65
Resolved episodes found the preceding week†, %	80	69
Ratio of distance-1 (observed/expected)	0.365	2.346
*P*‡	0.525	0.048
3 weeks		
Total episodes resolved*, %	51	65
Resolved episodes found the preceding week†, %	67	69
Ratio of distance-1 (observed/expected)	0.292	2.905
*P*‡	0.468	0.057
4 weeks (baseline)		
Total episodes resolved*, %	54	80
Resolved episodes found the preceding week†, %	63	56
Ratio of distance-1 (observed/expected)	0.264	2.676
*P*‡	0.243	0.025
8 weeks		
Total episodes resolved*, %	60	90
Resolved episodes found the preceding week†, %	57	50
Ratio of distance-1 (observed/expected)	0.420	2.179
*P*‡	0.229	0.014
17 weeks		
Total episodes resolved*, %	68	90
Resolved episodes found the preceding week†, %	50	50
Ratio of distance-1 (observed/expected)	0.478	2.101
*P*‡	0.617	0.033

*A potential infector was found.

†The number of incident-colonization episodes with a potential infector found during the previous week divided by the total number of incident-colonization episodes with a potential infector found over the 2-, 3-, 4-, 8- or 17-week windows.

‡Wilcoxon signed-rank paired test comparing observed vs. expected distances CPIs.

Extended-spectrum β-lactamase-producing *E*. *coli*, ESBL-EC; extended-spectrum β-lactamase-producing *K*. *pneumoniae*, ESBL-KP; CPI, close-proximity interaction.

### Simulations of the impact of control measures

**Fig 2:**
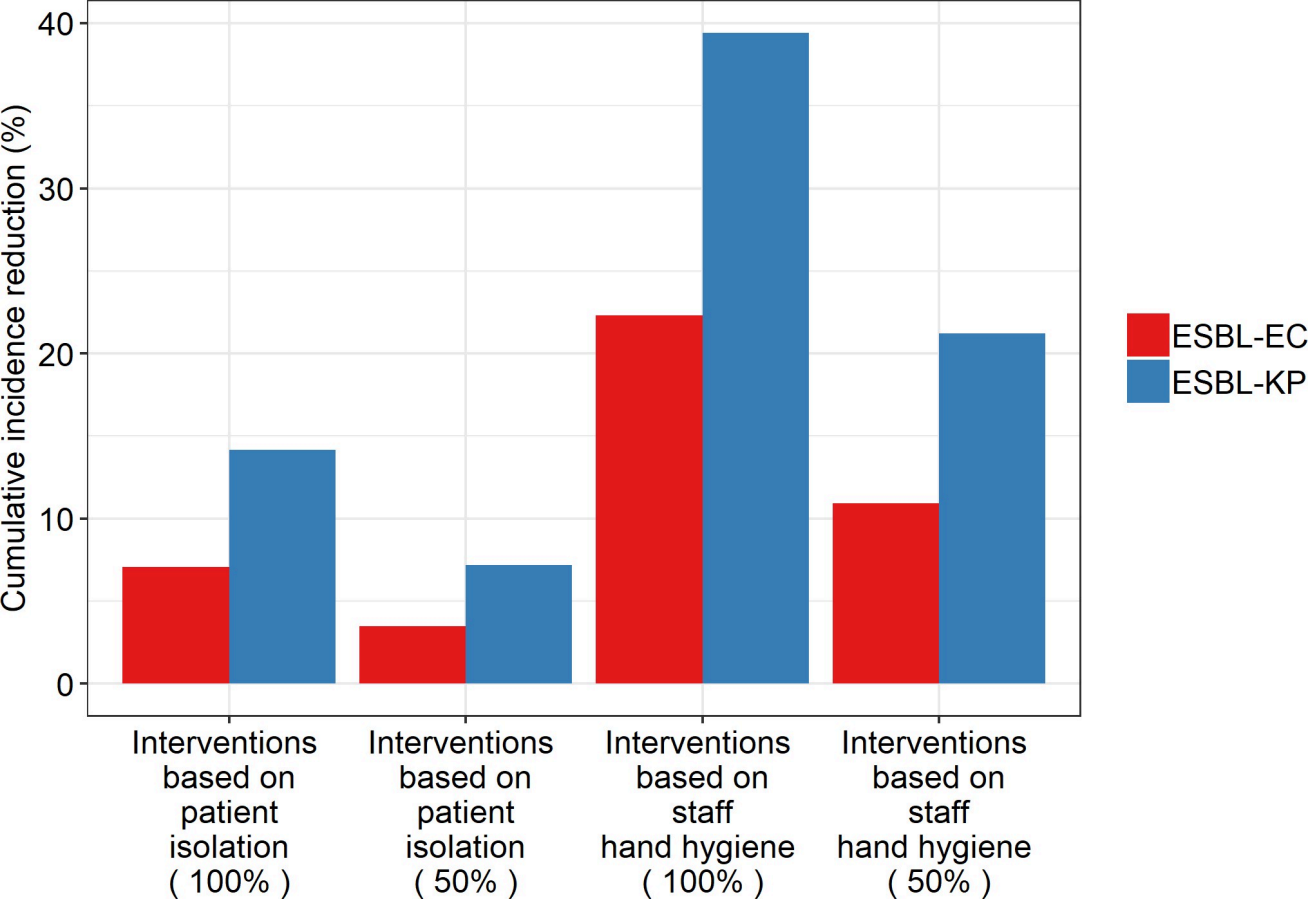
Predicted reduction in the cumulative incidence of ESBL-EC and ESBL-KP under 4 illustrative scenarios, using the mathematical model. For each scenario and each species, the percent reduction in the cumulative incidence compared to the baseline situation (without any control measure) is depicted (red: EC, blue: KP). Interventions based on patient case isolation correspond to a removal of 100% and 50% of patient-patient CPIs. Interventions based on staff hand hygiene correspond to a removal of 100% and 50% of patient-staff CPIs.

We used a mathematical model to assess the impact of our findings on the effect of control measures. We simulated transmission of an ESBL species in a 128-patient ward over 17 weeks. Without any control measure implemented, the model-predicted cumulative incidence over 4 months was 31% (40/128) patients for ESBL-EC and 19% (24 /128 patients) for ESBL-KP, in line with the weekly incidences that were observed during the i-Bird study (Table 2). The 4 explored illustrative scenarios, based on different levels of isolation and staff hand hygiene, all led to a reduction in incidence. For each control scenario, Fig 2 (S3 Fig) shows the relative reduction in the 4-month cumulative incidence for both ESBL-EC and ESBL-KP. All scenarios had a significantly larger impact for ESBL-KP than for ESBL-EC, with scenario 3 based on perfect staff hand hygiene being the most effective for both species. Indeed, scenario 3 led to a predicted 39% reduction of the ESBL-KP incidence and a 22% diminution in ESBL-EC incidence, while scenario 1 based on perfect patient isolation led to smaller respective reductions of 14% and 7%. As expected, scenarios 2 and 4 (imperfect compliance) were less effective, with scenario 2 achieving only 12% and 6% reductions of the ESBL-KP and ESBL-EC incidences, respectively.

## Discussion

For this study, contact patterns of patients and hospital staff were combined with weekly carriage data to trace the possible routes of resistant Enterobacteriaceae transmission in an LTCF. We found that the human contact network did not correspond to the spread of ESBL-EC, but supported that of ESBL-KP. Those findings suggested that transmission along CPIs is an important driver for ESBL-KP but that it is not the main driver in LTCF spread for ESBL-EC. This result is consistent with previous studies investigating the role of patient-to-patient transmission in the Enterobacteriaceae spread. Indeed, in a prospective cohort of patients admitted to a tertiary care hospital in the US, patient-to-patient transmission was shown to be an important cause of ESBL-KP but not ESBL-EC acquisition [23,24]. More recently, Gurieva et al. showed, using a modeling approach on data from 13 European intensive-care units, that ESBL-KP was over 3 times more transmissible than ESBL-EC [25]. Furthermore, Smit et al. revealed a high degree of cross-infection in the *K*. *pneumoniae* dynamics among a neonates unit in Cambodia [26]. Because LTCFs can be a hotspot for resistance acquisition [27,28], better understanding of resistant Enterobacteriaceae dissemination in these settings is an important step towards antibiotic-resistance control.

In our study, ESBL-EC and ESBL-KP were the dominant species among ESBL-producing Enterobacteriaceae, in accordance with the reported increase of these two species in Europe over the last years [3]. The importation and acquisition rates we observed (8% of admitted patients for importation rate and 0.66%/week acquisition rate for *E*. *coli*, 1% importation rate and 0.38%/week acquisition rate for *K*. *pneumoniae*) were globally higher than those recently reported in a French intensive care unit for ESBL-producing Enterobacteriaceae as a whole (8% importation rate and 0.29%/week acquisition rate) [29]. This may be due to differences in patient contact patterns and lengths of stay between intensive care units and LTCFs. We also observed a relatively high prevalence of ESBL-EC and ESBL-KP colonization among patients, which is consistent with previous findings in LTCFs [30]. The average duration of ESBL-producing Enterobacteriaceae carriage estimated from the collected data was 4 weeks (28.6 days, 95% CI: 19.9–37.3), which is shorter than most published estimates. However, these published estimates are scarce and highly variable; a European study estimated time after clearance for highly resistant Enterobacteriaceae to 42.6 days (95% CI: 9.7 - ∞), not far from our own estimate [31]. Because of the heterogeneous nature of *E*. *coli* including several strains with the ability to spread in different settings, acquisition of resistance in a LTCF could lead to a dissemination through the community. Indeed, LTCFs seem to be a reservoir for ESBL-EC resistance. In addition, ESBL-EC has been shown to have a high potential for dissemination through different types of healthcare facilities (including LTCFs and hospitals) and the community, as underlined by a study in which some strains were found in several distinct places such as administrative health areas, nursing homes, and community healthcare centers [32].

Our results suggest varied selection and dissemination patterns according to ESBL-producing species. On the one hand, most ESBL-KP acquisitions cases were observed in a specific ward, as opposed to broad dissemination of ESBL-EC throughout the entire LTCF. On the other hand, the diversity of resistance profiles was broader for ESBL-EC than for ESBL-KP, suggesting potentially higher diversity of circulating *E*. *coli* clones, which also agrees with other studies [33]. The 8-fold difference between observed *E*. *coli* and *K*. *pneumoniae* importation rates suggests that the majority of ESBL-EC carriers in our study acquired the bacteria in the community. This could explain the low portion of resolved ESBL-EC episodes found in our results [9]. Indeed, several patients sharing similar ESBL-EC isolates in the study could have been colonized through the community before hospitalization, leading to the identification of transmission candidates inside the LTCF but no potential infector over the contact network for these incident-colonization episodes. Another possible explanation for the low ESBL-EC transmission rate along CPIs is that ESBL-EC might have been acquired mostly through endogenous processes (e.g. plasmid exchange within the gut), after potential resistance acquisition from another species or the environment. Indeed, ESBL-EC is known to represent a resistance-gene reservoir in hospitals [34]. Antibiotic exposure could provide yet another explanation for unresolved incident-colonization episodes. Indeed, apparent incident-colonization episodes could in reality be unmasking of ESBL-producing Enterobacteriaceae carriage following antibiotic exposure. In order to investigate the impact of β-lactam use in our study, we calculated, for each incident colonization event, the delay between the last day of antibiotic use and the colonization event day. About 40% of patients involved in colonization events (15/37) had taken β-lactams before they were colonized which represents 27/55 episodes. Interestingly, slightly more of these 27 episodes occurred during the antibiotic treatment for ESBL-EC (5/16 episodes) than for ESBL-KP (3/11 episodes). The mean delay between β-lactam exposure and colonization was also shorter for ESBL-EC (10 days; 95% CI: 2–18) than for ESBL-KP (38 days; 95% CI: 7–68). These numbers suggest that antibiotic use should not have biased our results regarding ESBL-KP. In particular, at most 15% (3/20) of all ESBL-KP episodes occurred during an antibiotic treatment and could thus result from unmasking. Regarding ESBL-EC however, some apparent colonization events that could not be explained by the contact network may indeed turn out to be unmasking events following recent antibiotic use. These results were illustrated by the distribution of delays between antibiotic exposure and incident-colonization episode (S4 Fig). Finally, the definition of incident-colonization episodes, based on one negative swab followed by a positive one, was chosen because of the low number of episodes but did not account for imperfect swab sensitivity, potentially leading to false acquisition events. To ensure that this did not affect our results, a different definition of incident colonization requiring two observed negative swabs followed by a positive one was tested. These results were similar to those obtained with the baseline definition (S5 Fig), although the difference between observed and expected distances for ESBL-KP was not statistically significant at the 95% confidence threshold (P = 0.078), probably due to a lack of statistical power (8/10 episodes were resolved).

Herein, ESBL-EC and ESBL-KP were analyzed independently, unlike most previous studies in which ESBL-producing Enterobacteriaceae were considered globally, with no species. Although our approach enabled us to highlight the important dissemination differences between the two bacterial species, between-species gene exchanges within a host’s flora were not taken into account, probably contributing to the high unexplained portion of incident-colonization episodes with ESBL-EC. Future studies should be designed to specifically assess that question, which will require detailed data on multiple colonization.

In a previous i-Bird CPIs study [35], potential “superspreader” professions were identified among the hospital staff. Here, we built a generalized linear model (GLM) to explain the risk, for hospital workers, of having had contact with patients who acquired ESBL-EC or ESBL-KP in the 4 preceding weeks, using hospital worker profession (such as nurses, physicians, hospital porters and so on) as a predictor. This analysis was performed for the 9 isolates that were found to have been acquired by more than one patient (6 ESBL-EC and 3 ESBL-KP). Hospital worker profession was significantly associated with the predicted risk for 6 of these 9 isolates. Interestingly, hospital porters were found to be at increased risk of having had contact with incident cases for 3 out of 6 isolates (OR from 6.8 to 10.3). Being a reeducation staff increased the risk for 2 isolates (OR: 2.6, 95% CI: 1.11–6.05 and OR: 2.8, 95% CI: 1.06–7.49), as were nurses (OR: 2, 95% CI: 1.06–3.78 and OR: 2.3, 95% CI: 1.22–4.42). An in-depth investigation of the i-Bird data also allowed us to identify potential “superspreaders” among the hospital workers, although this was not associated to ESBL-EC or KP specifically. For instance, we found a hospital porter with whom all patients who acquired isolate 1 (an ESBL-EC), as well as all patients who acquired isolate 2 (an ESBL-KP), had had earlier contact. Results from the GLM analysis are available in S1 Table.

This study has several limitations.

First, neither the β-lactamase nor its coding gene were identified (or typed), leading us to propose an ad hoc definition of the most similar isolates based on their phenotypic resistance profiles. As suggested by the sensitivity analyses on this definition, its impact on our main results was low. However, we cannot be sure that similar antibiotic susceptibility profiles mean similar genetic strains. Since phenotypic information is not as precise as comparative genomic, there are multiple ways in which isolates with the same phenotype could belong to different lineages (especially since many resistance mechanisms are co-selected and carried on shared resistance elements). Conversely, different phenotypes could belong to the same lineage (e.g. horizontal gene transfer, alteration of expression of genes). Studies associating data on ESBL species transmission to genotypic and phenotypic information would probably give more information and will be the next step. Still, we believe that our study already provides some valid insight on ESBL-EC and ESBL-KP transmission in LTCFs. In particular, a part of ESBL-KP cases occurred during an outbreak localized in a single ward, the transmission hypothesis was in this case very likely, despite the lack of genotyping information. Moreover, there is a close relationship between *E*. *coli* genotype and phenotype as some studies show a good probability to predict phenotypic resistance through genotypic analysis for this species [36,37]. Hence, we can assume that using genotyping information might not affect qualitatively our main results on both ESBL-KP and ESBL-EC. A genetic-based definition of strains would probably be more restrictive and lead to more incident-colonization episodes than resistance phenotypes alone, but with fewer resolved episodes.

Second, CPIs capture all interactions at less than 1.5 m, which means that they do not necessarily involve a physical contact, especially when the CPI duration is short. Thus, it is possible that we captured some false positive "contacts", especially for patients who shared a room. In general, for most patients, it can be expected that those sharing a room had some contacts with the exception of persistent vegetative state (PVS) patients, who probably had little to no physical contacts with other patients, even while inhabiting the same room. Consequently, transmission between this category of patients might be more likely to occur indirectly, via HCWs or the endogenous route, and captured CPIs between them may not necessarily support bacterial spread. Therefore, it is important to note that a large part of the ESBL-KP–acquisition episodes that were resolved, had a potential infector at a distance-1 for PVS patients (7/13 cases were PVS patients), due to an outbreak of ESBL-KP in neurology ward 1 during the 4-month i-Bird study. More detailed observational data would be needed to fully understand this apparent patient-to-patient transmission of ESBL-KP to PVS patients. In contrast, only 1/35 ESBL-EC–acquisition episodes involved a PVS patient. Finally, because the i-Bird study took place in a LTCF, our results may not be generalizable to other healthcare settings, such as acute care hospitals. As mentioned earlier, resistance acquisition rates may be higher in LTCF, due to the specific dynamics of long-term hospitalization.

Our results have potentially important implications in terms of infection control. Indeed, for ESBL-EC, the most frequently observed case-to-potential infector path was distance-2, with mainly hospital staff intermediaries. Although this might partially reflect the fact that only patients’ swabs were tested for Enterobacteriaceae, the observed pattern of contacts in our LTCF (with frequent patient-patient interactions) suggests that many patient-to-patient CPIs did not result in ESBL-EC transmission. That deduction implies that contact precautions, which are currently the most commonly implemented control measure to prevent ESBL-EC spread [38], may not be fully effective. Our results are consistent with earlier analyses and observations [34,39,40]. Tschudin-Setter et al. found that the transmission rate of ESBL-EC was similar before and after discontinuation of contact precautions in an acute care hospital [41]. These results are consistent with those of a 5-year observational study performed by Zahar et al. in two French hospitals, in which there was no evidence of an impact of improved contact precautions on the incidence of ESBL-EC [39]. Finally, in a large-scale American study, Goto et al. found a larger reduction in *Klebsiella spp* bacteremia rates than in *Escherichia coli* bacteremia rates after the implementation of the methicillin-resistant *Staphylococcus aureus* “Prevention and Initiative” program, which involved contact precautions and hand hygiene [42]. To further investigate this question, we developed a compartmental model of ESBL-EC or KP spread within a LTCF, and simulated two illustrative control measures (patient isolation and staff hand hygiene) with parameters estimated from the study data. Our analyses showed that reducing CPIs, especially between patient and staff (through perfect hand hygiene), might decrease the incidence of both ESBL-EC and ESBL-KP, but with a significantly larger reduction for the latter (Fig 2). Similar results were obtained under various scenarios regarding intervention compliance (isolation with 75%, 50% or 25% of patient-patient contacts removed, staff hand hygiene with 75%, 50% or 25% of patient-staff contacts removed) even if the carriage duration was extended (S3 and S6 Figs). Those findings confirmed that contact-precaution strategies are bound to be highly effective at controlling ESBL-KP, while additional measures such as environmental decontamination or antimicrobial stewardship, might be needed for ESBL-EC.

This study, by jointly analyzing longitudinal ESBL-producing Enterobacteriaceae carriage data with CPI records using radio-frequency identification technology, contributes to our understanding of the dynamics of ESBL-EC and ESBL-KP spread. We showed that CPI information is useful to track ESBL-KP transmission among patients, but not for ESBL-EC. That difference sheds light on the fact that transmission patterns vary according to the species and that species-adapted strategies are needed when aiming to effectively control antibiotic resistance.

## Materials and methods

### Epidemiological data: The i-Bird study

The i-Bird study was conducted at the Berck-sur-Mer rehabilitation center from May 1 to October 25, 2009, with the first 2 months serving as a pilot phase. Rehabilitation centers often require long inpatient periods, unlike acute-care facilities. All participants, 329 patients and 263 hospital staff, wore a badge-sized wireless sensor to record CPIs throughout the study. During that period, rectal swabs were collected weekly from patients to test for Enterobacteriaceae carriage. On average, participating patients were swabbed on 64% of their weeks of stay in the LTCF. In most cases, the delay between two swabs was 7 days (S7 Fig).

Hospital staff included all health professionals: healthcare workers (HCWs, including nurses, auxiliary nurses, nurse managers and student nurses), reeducation staff (physical and occupational therapists), ancillary hospital staff, physicians, hospital porters, logistic, administrative and animation staff. The LTCF was subdivided into 5 wards, corresponding to medical specialties: neurological rehabilitation (ward 1, 2 and 4), obesity care (ward 3) and geriatric rehabilitation (ward 5).

### CPI description

Every 30 s, each wireless sensor recorded the identification number of all other sensors within a radius of less than 1.5 m and time of interaction. Over 4 months, from July to the end of October, 2,740,728 such distinct CPIs were recorded for 592 persons. This CPI-network was then aggregated at the daily level. To describe CPIs, we used two indicators: the number of daily distinct CPIs of a given individual and the daily cumulative duration of CPI between two individuals. The first indicator represents the total number of unique individuals met over a day. Detailed definitions of these indicators are provided in an earlier paper [35].

### Microbiological data

Rectal swabs were collected weekly from patients. Briefly, swabs were placed in Stuart’s transport medium (500μL; Transwab, Medical 90 Wire and Equipment). Each 100-μL aliquots was plated on selective media for ESBL isolation. The rest of the suspension was then stored at −80°C for further use. Antimicrobial susceptibility testing was done each week for ESBL-producing Enterobacteriaceae, in accordance with national recommendations [43].

### Definitions of carriage

For this study, we independently investigated the spread of two distinct Enterobacteriaceae species: ESBL-EC and ESBL-KP.

#### Most similar isolate definition

Most similar isolates were defined based on their species characterization and phenotype-resistance profile. Because all bacteria were ESBL-producing Enterobacteriaceae, we focused on only 12 antibiotics, including 5 aminoglycosides (kanamycin, gentamicin, tobramycin, netilmicin, and amikacin), 4 fluoroquinolones (nalidixic acid, ofloxacin, levofloxacin, and ciprofloxacin), co-trimoxazole, tetracyclin and fosfomycin. We exclude ESBL selective antibiotics. Clustering analyses confirmed that the resistance profiles to these 12 antibiotics allowed to define clusters of isolates (Section S1 and S8A Figs). In addition, antibiotic susceptibility profiles of isolates were clearly differentiated, with one group dominated by ESBL-EC and the other, more heterogeneous group, had a majority of ESBL-KP (S8B Fig).

Based on those results, we assumed that two ESBL-producing Enterobacteriaceae isolates were the most similar when they belonged to the same species and had the same resistance-sequence status to the 12 selected antibiotics (allowing for R–I or S–I differences for each antibiotic).

#### Definition of prevalence and incidence

Average weekly prevalence and incidence were determined over the 4-month study period for each ESBL species and each isolate (Section S2). Weekly prevalences were defined as the proportion of colonized patients among swabbed patients during each week of the study period. For weeks with less than 10 swabbed patients the prevalence and the incidence were considered as missing data because of the high uncertainty level in weeks with fewer swabs. We defined an incident-colonization episode for a given week as the isolation from a patient of an isolate or an ESBL species that had not been found in the same patient the preceding week. The weekly incidence was defined as the number of patients with incident-colonization episodes for a given week, divided by the number of patients not colonized by the same ESBL species or by the most similar isolate in the preceding week.

#### Definition of “cases”, “transmission candidates” and “potential infectors”

A “case” was defined as a patient with an incident-colonization episode. A “transmission candidate” for a case was defined as a patient who carried the most similar isolate to the case over the preceding 4 weeks (which was the average duration of ESBL carriage in this study). Finally, a case’s “potential infector” was a “transmission candidate” for whom a path linking to the case existed on the CPI-network over the preceding 4 weeks. Thus, potential infectors for a case refer to all individuals who could be at the origin of the transmission to the case through the CPI-network. Among all potential infectors, the closest potential infector was the one with the shortest distance to the incident case. When at least one potential infector was found for a given incident case, this case was classified as “resolved”, otherwise it was “unresolved”.

#### Definition of importation and acquisition rates

The importation rate represents the proportion of all admitted patients over the 4 months of study who were colonized at admission. The weekly acquisition rate is computed as the number of incident-colonization episodes among included patients over the 4 months of study, divided by the total number of included patients over this period and by the study duration, in weeks (Section S3).

### Assessment of the impact of CPIs on transmission of antibiotic resistant bacteria

As described previously [21], the length of the shortest CPI-supported transmission path allows measurement of the link between CPIs and bacterial carriage. We tested whether the observed distances between cases and their closest potential infector in the CPI network were different from those expected under the null hypothesis of independence between CPIs and carriage data.

#### Observed distance

**Fig 3:**
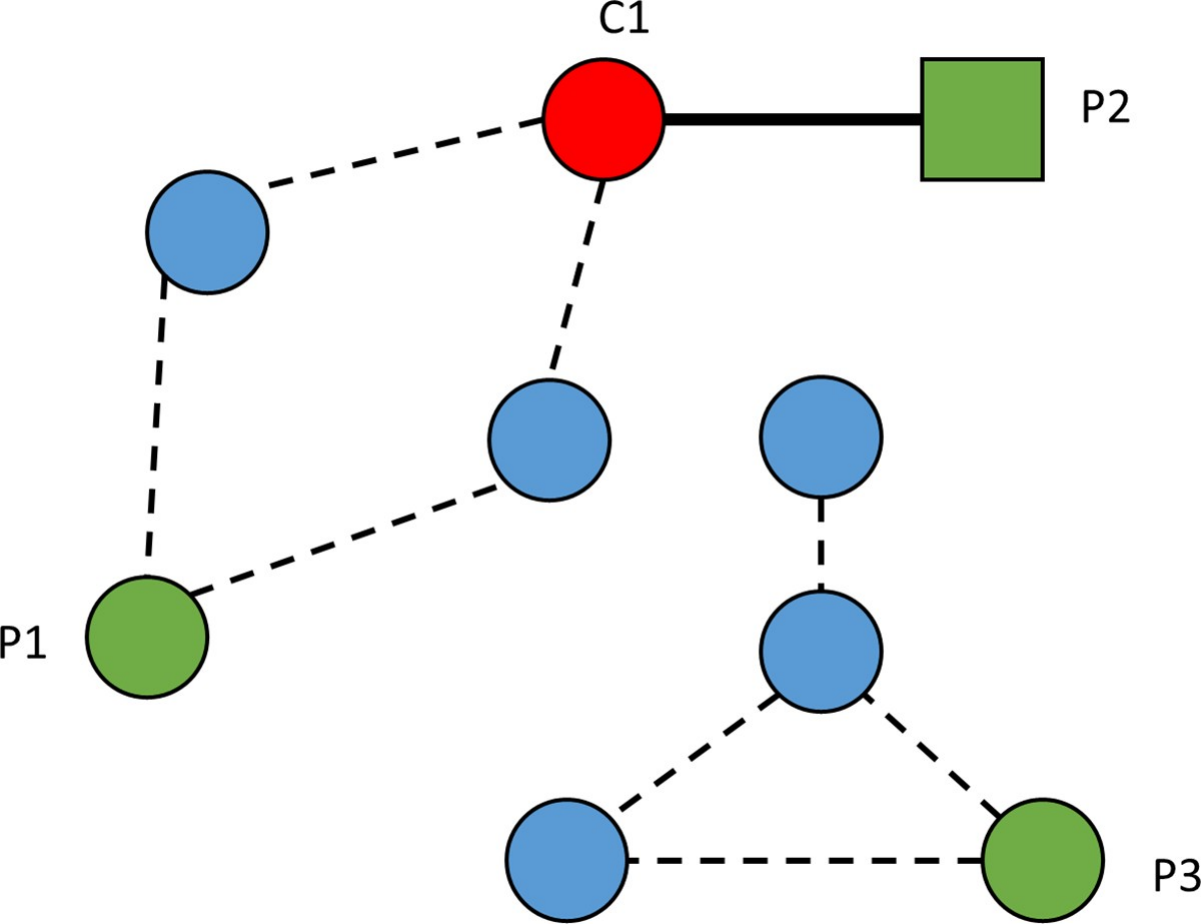
Description of close-proximity interactions (CPIs) and determination of their distances through combined weekly carriage data and CPI-network plots. Circles and rectangles (nodes) represent patients. The red circle C1 represents a case with an incident-colonization episode. Green circles and rectangles P1, P2, P3 represent transmission candidates, who were colonized with the most similar isolate during the preceding 4 weeks. Patients P1 and P2 are potential infectors, as they are connected to the incident case via edges in the CPI network. The closest potential infector is patient P2 (represented by a rectangle). The distance is 1 because no intermediary is present between C1 and P2 (solid black line). Blue circles represent individuals susceptible to colonization.

For each incident-colonization episode, the observed distance was determined as follows: first, we looked for candidate transmitters carrying the most similar isolate during the preceding weeks. Then, for each candidate, we computed the shortest CPI path (i.e. number of edges) to the case over the last 4 weeks and retained the closest potential infector as the one with the shortest CPI path to compute the distance (Fig 3).

#### Expected distance

For each incident-colonization episode, the expected distance under the null hypothesis was computed through Monte Carlo simulations (Section S4). We randomized all carriage data among the network nodes over the preceding 4 weeks. For each incident-colonization episode, 200 replicates of permutated carriage statuses were obtained. In each permutated dataset, the shortest CPI path was computed as above. The expected distance was then computed by averaging all the shortest lengths of CPI path to this colonization episode.

#### Statistical test

Finally, for all incident-colonization episodes collected, expected distances and observed distances were compared using the Wilcoxon signed rank paired test.

### Sensitivity analyses

To assess the influence of assumptions regarding the most similar isolate definition and colonization duration on the results, CPI-transmission analyses were repeated with different definitions and investigation periods. Five outcome indicators were analyzed: (1) the number of incident-colonization episodes; (2) the percentage of resolved incident-colonization episodes (for which potential infectors had been found); (3) the percentage of resolved episodes over the preceding week, which was calculated as the number of incident-colonization episodes for which a potential infector had been found at week 1 divided by the total number of incident-colonization episodes with a potential infector found (Section S5); (4) the ratio of observed versus expected distance-1; and (5) the *P*-value obtained from the Wilcoxon signed rank paired test between observed and expected distances.

First, we compared the five outcome indicators for the results obtained using the initially described most similar isolate definition (baseline) in the analysis with those derived with a stricter definition. In the latter, S–I and I–R differences were taken into account, meaning that for a given incident case, transmission candidates were those carrying the most similar isolates with the exact same phenotypic resistance profile, as opposed to the less strict baseline definition which allowed those I–S and I–R variations.

Then, the impact of the period during which transmission candidates were sought was examined. We repeated the analysis for 2, 3, 4, 8 and the entire 17-weeks study period. The same five indicators were assessed, except for the number of incident-colonization episodes which did not vary according to the considered period duration.

### Deterministic model and simulation

We built a susceptible–colonized model of a 128-patient LTCF (mean number of patients per week over the study period), in which susceptible (non-colonized) patients could acquire ESBL-producing Enterobacteriaceae following contact with a colonized patient, at a rate *β*_*B*_ for bacteria *B*. *β*_*B*_ was computed as the product of the pathogen-specific per-contact transmission probability (*p*_*B*_) by the weekly distinct number of patient-to-patient CPIs at a distance-1 or distance-2 (*c*_*P*_) observed in the i-Bird CPI-network. Susceptible patients could also become colonized with bacteria *B* at a rate *ν*_*B*_ through the environment or the endogenous route, as previously proposed by Bootsma et al. [44]. *ν*_*B*_ was computed as the product of the proportion of incident-colonization episodes for which a potential infector was not found at a distance equal or less than 2 (1 - *τ*_*B*_), by the weekly incidence rate (*i*_*B*_) of the pathogen observed in the i-Bird data. Colonized patients returned to the susceptible state at a rate *γ*_*B*_, equal in average to 1/*D*_*B*_, where *D*_*B*_ was the duration of bacteria *B* colonization. The model was parameterized for ESBL-EC or ESBL-KP independently. All parameter values were directly taken from the observed i-Bird study data, except for the per-contact transmission probabilities *p*_*B*_, which were computed so that the predicted steady-state colonization prevalence reproduced the observed data (Table 1). More model details, including model equations and details of baseline parameter computation, are provided in Section S6.

We compared the impacts of two simple illustrative control measures with varying levels of compliance, leading to 4 scenarios: scenarios 1 and 2, in which a portion of patient-patient CPIs was removed to simulate patient contact isolation (scenario 1: 100%, scenario 2: 50%); and scenarios 3 and 4, in which a portion of patient-staff CPIs were removed to simulate staff hand hygiene (scenario 3: 100%; scenario 4: 50%). For each scenario, the mean number *c*_*P*_ of weekly CPIs under a distance-2 was re-computed from the i-Bird data. The corresponding values are provided in Table 4 and Table 5. All statistical analyses were performed with R version 3.3.2 (http://www.r-project.org/).

**Table 4:** Fixed model parameters.

Model parameter	Symbol	Value	Source
Per-contact probability of bacterial transmission			
*E*. *coli*	*P*_*ec*_	0.001	Computed (SI S6)
*K*. *pneumoniae*	*P*_*kp*_	0.003	
Duration of bacterial colonization (week)			
*E*. *coli*	*D*_*ec*_	5.9	Estimated from i-Bird data
*K*. *pneumoniae*	*D*_*kp*_	3.2	
Proportion of incident-colonization episodes with a potential infector at a distance ≤ 2			
*E*. *coli*	*τ*_*ec*_	0.51	Estimated from i-Bird data
*K*. *pneumoniae*	*τ*_*tk*_	0.75	
Weekly incidence rate			
*E*. *coli*	*i*_*ec*_	0.0196	Estimated from i-Bird data
*K*. *pneumoniae*	*i*_*kp*_	0.0115	
Weekly rate of colonization from the environment or the endogenous route: ν = *i*×(1 –*τ*)			
*E*. *coli*	*ν*_*ec*_	0.0096	Computed
*K*. *pneumoniae*	*ν*_*kp*_	0.0029	
No. of patients	*N*	128	Estimated from i-Bird data

**Table 5:** Scenario-related parameters.

		Intervention scenarios				
		Baseline	Based on patient case isolation		Based on staff hand hygiene	
Scenario-related parameter	Symbol	None	100%	50%	100%	50%
No. of distinct CPIs/week at CPI distance ≤2	*c*	81.4	73.8	77.6	55.2	69.6

### Ethics

All authorizations were obtained in accordance with French regulations regarding medical research and information processing. All French IRB-equivalent agencies accorded the i-Bird program official approval (CPP 08061; Afssaps 2008-A01284-51; CCTIRS 08.533; CNIL AT/YPA/SV/SN/GDP/AR091118 N°909036). Signed consent by patients and staff was not required according to the French Ethics Committee to which the project was submitted.

## Supporting information

S1 Text Resistance profiles of the acquired isolates and clustering description. (DOCX)

S2 Text Prevalence and incidence definition. (DOCX)

S3 Text Importation and weekly acquisition rate. (DOCX)

S4 Text Pseudo-code for the calculation of the network-associated distance between an incident-colonization episode and potential infector. (DOCX)

S5 Text Calculation of the percentage of resolved episodes over the preceding week. (DOCX)

S6 Text Mathematical model of bacterial spread within a LTCF: Model representation, equations, computation of the parameters from the i-Bird data and steady state analysis. (DOCX)

S1 Fig Summary of the numbers of colonization, admission, acquisition over the 4-months period. (TIF)

S2 Fig Number of episodes with a majority of patients or hospital staff intermediaries. Here, only incident-colonization episodes with a distance-2 to their potential infector are considered. The portions of these episodes in which there is a majority of patients and hospital staff are depicted for (A) ESBL-EC and (B) ESBL-KP. These portions are compared between observed (light grey) and randomly permutated data (dark grey).
(TIFF)

S3 Fig Cumulative incidence reduction from eleven scenarios of the ESBL-EC and ESBL-KP models. Percentage on the y-axis corresponds to reduction of the cumulative incidence compared to the baseline (scenario with no control measure). In red, percentage of cumulative incidence reduction of ESBL-EC and blue ESBL-KP. Intervention based on patient isolation correspond to a removal of 100%, 75%, 50% and 25% of patient-patient CPIs. Intervention based on staff hand hygiene correspond to a removal of 100%, 75%, 50% and 25% of patient-staff CPIs_._
(TIFF)

S4 Fig Illustration of the time interval between the last antibiotic use and incident-colonization episodes. The distribution of the delay between the last day of antibiotic use and the colonization event day is depicted for both ESBL-EC and ESBL-KP. Delays were shorter for ESBL-EC than for ESBL-KP, leading to fewer swabs in the interval.
(TIFF)

S5 Fig Distribution of distances between acquisition cases and their closest potential infector with a second definition of incident-colonization episode. Incident-colonization episodes were defined as two negatives swabs followed by one positive swab for a given ESBL-EC or ESBL-KP isolate. Comparison between observed data (light blue) and random permutated data (dark blue). For each incident-colonization case, potential infectors were selected as the closest in the CPI-network of all candidates sharing the most similar isolate as the case in the preceding 4 weeks. (A) ESBL-EC distribution. (B) ESBL-KP distribution. Here distance is the number of edge between two individuals in the network. Because of the few episodes resolved (14/25 and 8/10 for ESBL-EC and ESBL-KP respectively), expected distances were computed as the average of distances obtained from 500 instead of 200 simulations using randomly permutated carriage data.
(TIF)

S6 Fig Sensitivity analysis of carriage duration in the mathematical model analysis. Different durations of carriage were used: 11.73, 17.60 and 23.47 weeks for ESBL-EC and 6.49, 9.73 and 12.97 weeks for ESBL-KP. The percentage on the y-axis corresponds to the reduction of the cumulative incidence compared to the baseline scenario (with no control measure), for ESBL-EC (in red) or ESBL-KP (in blue). Interventions based on patient isolation correspond to a removal of 100% or 50% of patient-patient CPIs. Intervention based on staff hand hygiene correspond to a removal of 100% or 50% of patient-staff CPIs. The assumed duration of carriage for ESBL-EC and ESBL-KP is mentioned in the grey rectangle at the right of each graph. No matter the duration of carriage, reduction of cumulative incidence is more important for ESBL-KP than for ESBL-EC and more efficient when the intervention is based on staff hand hygiene.
(TIF)

S7 Fig Distribution of the delay between consecutive patient’s swabs. The time interval between two swabs was mostly at 7 days.
(TIFF)

S8 Fig Resistance profile of ESBL-producing Enterobacteriaceae detected in patients over the study period. (A), Each row represents an isolate identified during the study. Each column represents the phenotype sequence in terms of antibiotic resistance level to each of the 12 tested antibiotics. R, resistant (dark blue). I, intermediate (blue). S, susceptible (light blue) and U unknown (black). Tested antibiotics were penicillins (aminoglycosides (kanamycin (K), gentamicin (GM), tobramycin (TM), netilmicin (NET), amikacin (AN)), fluoroquinolones (nalidixic acid (NA), ofloxacin (OFX), levofloxacin (LVX), ciprofloxacin (CIP)), co-trimoxazole (SXT), tetracyclines (TE) and fosfomycin (FOS). The dendrogram was built from the distances between two phenotype profiles for the 12 antibiotics. (B) The same data is represented with characterization of the species. Blue: ESBL-EC, red: ESBL-KP and green: resistance sequences found in both species. Rectangle heights correspond to the number of individuals each profile was observed in.
(TIF)

S9 Fig Representation of the model. *S* and *C* are the susceptible and colonized compartments. *β_B_* is the weekly effective contact rate, *N* is the total number of patients within the LTCF, *ν_B_* is the weekly colonization-acquisition rate via the endogenous route or the environment and *γ_B_* is the decolonization rate of bacteria *B*.
(TIF)

S1 Table Hospital worker professions associated with having contact with patients who acquired ESBL-EC or ESBL-KP. Generalized linear model performed on 6 isolates involved in the acquisition of more than one patient. P-values were computed with a likelihood ratio test. “Other” gathers administrative, logistic and animation staff.
(DOCX)
