# Supplementary material for: Close proximity interactions support transmission of ESBL-K. pneumoniae but not ESBL-E. coli in healthcare settings
Source: PLoS Comput Biol. 2019 May 30;15(5):e1006496. doi: 10.1371/journal.pcbi.1006496 (PMC6542504; doi:10.1371/journal.pcbi.1006496)
Supplement: S1 Text — (DOCX) [file pcbi.1006496.s001.docx]

**S1 text: Resistance profiles of the acquired isolates and clustering description**

First, the resistance profiles to 28 antibiotics were determined for each identified Enterobacteriaceae from the collected swabs as a sequence of *n* = 28 S, I or R (susceptible, intermediate or resistant, respectively) providing the resistance status to the 28 antibiotics. Because of the wide variability of those profiles among ESBL-producing Enterobacteriaceae and the phenotype detection limits, we clustered bacteria according to their phenotypic profiles. A phenotype distance between two similar isolates was defined by counting the number of R–S mismatches between the two, ignoring S–I or I–R mismatches.

The distances P_i_ = ${(X_{k}^{\left( i \right)})}_{k=1\ldots n}$ and P_j_= ${(X_{k}^{\left( j \right)})}_{k=1\ldots n}$ were defined for two resistance profiles with *n* the number of antibiotic resistance phenotypes of the sequence:

|  | $D(P_{i,}P_{j})= \sum_{k=1}^{n} d_{k}, with d_{k}= \left\{ \begin{aligned} 1 \\ 0 \end{aligned}{\text{if} (X_{k}^{\left( i \right)}=S and X_{k}^{\left( j \right)}=R) \text{or if }{(X}_{k}^{\left( i \right)}=R and X_{k}^{\left( j \right)}=S) \atop otherwise} \right.$ | (1) |
| --- | --- | --- |

The clustering analysis used the complete linkage method of the function hclust from R version 3.3.2 (<http://www.r-project.org/>) . Isolates clusters were based on the calculated distances.

We applied a simplified definition of the sequence and differentiated clusters based on a 12-antibiotic resistance profile. That simplified profile includes 5 aminoglycosides (K for kanamycin, GM for gentamicin, TM for tobramycin, NET for netilmicin, and AN for amikacin), 4 fluoroquinolones (NA for nalidixic acid, OFX for ofloxacin, LVX for levofloxacin, and CIP for ciprofloxacin), co-trimoxazole (SXT), tetracycline (TE) and fosfomycin (FOS). With the aim of characterizing similar isolates, bacteria identified as ESBL-EC or ESBL-KP during the study period were also clustered according to this 12-antibiotic–resistance phenotype (SI Appendix, Fig. S8A).

Indeed, in Fig. S8A, different populations may be observed, according to their resistance profiles. In particular, on the upper left part of the figure, ESBL bacteria resistant to aminoglycosides and susceptible to fluoroquinolones on the one hand, and ESBL bacteria susceptible to both antibiotics, appear to form separate groups. In the bottom right side, more heterogeneity is observed. In Fig. S8B, the upper left and bottom right sides can be separated according to the species, with a majority of ESBL-EC on the upper left side and ESBL-KP mostly on the bottom right side. 8 sequences among 96 were shared by ESBL-EC and ESBL-KP
