# Supplementary material for: Close proximity interactions support transmission of ESBL-K. pneumoniae but not ESBL-E. coli in healthcare settings
Source: PLoS Comput Biol. 2019 May 30;15(5):e1006496. doi: 10.1371/journal.pcbi.1006496 (PMC6542504; doi:10.1371/journal.pcbi.1006496)
Supplement: S2 Text — (DOCX) [file pcbi.1006496.s002.docx]

**S2 text: Prevalence and incidence definition**

Prevalence and incidence were determined by averaging the weekly values over the *W* weeks of the study period. Let *P* be the total number of patients included in the study. For each week *w* (in 1...*W*) and any patient *p* (in 1...*P*), let *P_wp_* be an indicator of presence within the LTCF of patient *p* during week *w* (*P_wp_* = 1 if patient *p* was present), and let *C_wp_* be an indicator of colonization for patient *p* on week *w* (*C_w p_* = 1 if patient *p* was colonized). Then the weekly prevalence and incidence during week *w* (in 1…*W*) can be computed as:

$$Prev\left( w \right)=\frac{\sum_{p=1}^{P} P_{wp}\times C_{wp}}{\sum_{p=1}^{P} P_{wp}}$$

$$Inc\left( w \right)=\frac{\sum_{p=1}^{P} P_{w-1,p}\times{(1-C}_{w-1,p})\times P_{wp}\times C_{wp}}{\sum_{p=1}^{P} P_{w-1,p}\times{(1-C}_{w-1,p})}$$

The average weekly prevalence and incidence over the study period can be computed as:

$Prevalence= \frac{1}{W}\times\sum_{w=1}^{W} \frac{\sum_{p=1}^{P} P_{wp}\times C_{wp}}{\sum_{p=1}^{P} P_{wp}}=\frac{\sum_{w=1}^{W} Prev(w)}{W}$ (2)

$Incidence=\frac{1}{W}\times\sum_{w=2}^{W} \frac{\sum_{j=1}^{P} P_{i-1,j}\times\left( 1-C_{i-1,j} \right)\times{P_{i,j}\times C}_{i,j}}{\sum_{j=1}^{P} P_{i-1,j}\times\left( 1-C_{i-1,j} \right)} = \frac{\sum_{w=2}^{W} Inc(w)}{W}$ (3)
