# Supplementary material for: Close proximity interactions support transmission of ESBL-K. pneumoniae but not ESBL-E. coli in healthcare settings
Source: PLoS Comput Biol. 2019 May 30;15(5):e1006496. doi: 10.1371/journal.pcbi.1006496 (PMC6542504; doi:10.1371/journal.pcbi.1006496)
Supplement: S3 Text — (DOCX) [file pcbi.1006496.s003.docx]

**S3 text: Importation and weekly acquisition rate**

Global importation and acquisition rates were calculated over the *W* weeks of the study period as follows:

$Importation Rate= \frac{\sum_{w=1}^{W} {CA}_{w}}{\sum_{w=1}^{W} A_{w}}$ (4)

where *CA_w_* is the number of colonized participating patients admitted during week *w*, and *A_w_* is the number of participating patients admitted during week *w*.

$weekly Acquisition Rate= \frac{1}{W}\times\frac{1}{P}\times\sum_{w=1}^{W} \sum_{p=1}^{P} P_{w-1p}\times\left( 1-C_{w-1p} \right)\times{P_{wp}\times C}_{wp}$ (5)

where *P* is the total number of patients included in the study, and for any week *wp* (in 1...*W*) and any patient *p* (in 1...*P*), *P_wp_* is an indicator of presence within the LTCF of patient *p* during week *w* (*P_wp_* =1 if patient *p* was present), and *C_wp_* is an indicator of colonization for patient *p* during week *w* (*C_wp_* =1 if patient *p* was colonized).
