# Supplementary material for: Close proximity interactions support transmission of ESBL-K. pneumoniae but not ESBL-E. coli in healthcare settings
Source: PLoS Comput Biol. 2019 May 30;15(5):e1006496. doi: 10.1371/journal.pcbi.1006496 (PMC6542504; doi:10.1371/journal.pcbi.1006496)
Supplement: S4 Text — (DOCX) [file pcbi.1006496.s004.docx]

**S4 text: Pseudo-code for the calculation of the network-associated distance between an incident-colonization episode and potential infector**

**For each incident-colonization episode *i* = 1 to Ncases do**

1. *Identify the episode*

Let the concerned patient as *p^i^*, the date as *w^i^* and the microbiological result as *m^i^*

1. *Find all swabs from individuals other than* p^i^ *taken during the time window [*w^i^*–*W*,* w^i^*–*1*]*

Tab = table of patient IDs/swab dates/microbiological results

**# To compute the observed distance**

1. *Find all transmission candidates in Tab*

For each swab in Tab

If microbiological result (swab) = *m^i^* Add swab to CandidateTab

1. *Compute the distance* dt *from the episode to each transmission candidate* t

For each transmission candidate *t* in Candidate Tab

*dt* = 1

While [(found = FALSE) and (full = FALSE)] repeat

IDlist = list of all patient IDs with network links of length *dt* to patient *p^i^* in the time window [*w^i^*–*W*, *w^i^*–1]

If length of the IDlist = total number of individual in the network do full = TRUE

If *t* is in IDlist do Set found = TRUE

Else do *dt* = *dt* + 1

1. *Determine the distance to the closest potential infector*

Compute *d* as the minimum of all *dt*

**# To compute the expected distance under H0**

**Do *n* times**

1. *Shuffle the microbial data*

Randomly shuffle the last column of Tab

1. *Perform steps 2 to 4 to compute the episode’s distance to its closest potential infector*

- **Compute *d_exp_* as mean of the *n* distances**
