# Supplementary material for: Close proximity interactions support transmission of ESBL-K. pneumoniae but not ESBL-E. coli in healthcare settings
Source: PLoS Comput Biol. 2019 May 30;15(5):e1006496. doi: 10.1371/journal.pcbi.1006496 (PMC6542504; doi:10.1371/journal.pcbi.1006496)
Supplement: S5 Text — (DOCX) [file pcbi.1006496.s005.docx]

**S5 text: Calculation of the percentage of resolved episodes over the preceding week**

The percentage of resolved episodes during the preceding week represents the proportion of episodes with a potential infector found during the preceding week among all incident-colonization episodes that were detected during the entire study detection period. We calculated this percentage as follows

$Resolved episodes= \frac{E_{CP, 1}}{\sum_{w=1}^{W} E_{CP,w}}$ (6)

Where *W* is the total number of weeks in the study, $E_{CP,1}$ is the number of incident-colonization episodes with a potential infector found during the preceding week and $E_{CP,i}$ is the number of incident-colonization episodes with a potential infector found during week *w*.
