# Supplementary material for: Close proximity interactions support transmission of ESBL-K. pneumoniae but not ESBL-E. coli in healthcare settings
Source: PLoS Comput Biol. 2019 May 30;15(5):e1006496. doi: 10.1371/journal.pcbi.1006496 (PMC6542504; doi:10.1371/journal.pcbi.1006496)
Supplement: S6 Text — (DOCX) [file pcbi.1006496.s006.docx]

**S6 text: Mathematical model of bacterial spread within a LTCF: model representation, equations, computation of the parameters from the i-Bird data and steady state analysis.**

***Notations***

Let *C(t)* be the number of patients within the LTCF colonized with bacteria *B* at time *t*, and *S(t)* the number of patients not colonized with that bacterium, and therefore susceptible to acquire its colonization. At all times: *S(t)*+*C(t)* = *N*, the total number of patients within the LTCF.

Let *β_B_* be the weekly effective contact rate, computed as:

*β_B_ =* $p_{B}\times c_{P}$

where $c_{P}$is the per-patient average weekly number of CPIs at a distance ≤ 2, and $p_{B}$is the per-contact transmission probability of bacteria *B*.

Let ${}_{B}$ be the weekly colonization-acquisition rate via the endogenous route or the environment, computed as:

${}_{B}= i_{B}\times\left( 1-\tau_{B} \right)$

where $i_{B}$ is the observed weekly incidence rate of bacteria *B* and $\tau_{B}$ is the proportion of cases of incident-colonization with bacteria *B* for which a potential infector was found at a distance ≤ 2.

Let $\gamma_{B}$be the decolonization rate of bacteria *B*, computed as:

$$\gamma_{B}=1/D_{B}$$

where $D_{B}$ is the average duration (in weeks) of colonization with bacteria *B*, estimated as the ratio of the average prevalence of colonization with bacteria *B* by the average weekly incidence of colonization with bacteria *B*.

***Model equations***

Time changes in *S(t)* and *C(t)* are driven by the following differential equations:

$$\left\{ \begin{aligned} \frac{dS}{dt}=-\beta_{B}\times S\times\frac{C}{N}-{}_{B}\times S+\gamma_{B}\times C \\ \frac{dC}{dt}=\beta_{B}\times S\times\frac{C}{N}+{}_{B}\times S-\gamma_{B}\times C \end{aligned} \right.$$

***A schematic representation of the model is presented on Fig S9***

***Computation of*** $\boldsymbol{p}_{\boldsymbol{B}}$

The steady-state values of *S* and *C*, denoted as *S^*^* and *C^*^*, verify the following equation:

$\beta_{B}\times S^{*}\times\frac{C^{*}}{N}+{}_{B}\times S^{*}-\gamma_{B}\times C^{*}=0$ (7)

Let *s^*^* and *c^*^* be the steady-state proportions of susceptible and colonized patients within the LTCF:

*s^*^ = S^*^/N* and *c^*^ = C^*^/N*.

Then it can be inferred from (7) that:

$$\beta_{B}=\frac{\gamma_{B}}{s^{*}}-\frac{{}_{B}}{c^{*}}$$

And as *β_B_* = $p_{B}\times c_{P}$, the per-contact probability of bacteria *B* transmission *p_B_* can be computed as:

$$p_{B}=\frac{1}{c_{P}}\times\left( \frac{\gamma_{B}}{s^{*}}-\frac{{}_{B}}{c^{*}} \right)$$

***Numerical application for ESBL-EC***

Based on the i-Bird CPI data, the baseline per-patient average weekly number of CPIs at a distance ≤ 2 is $c_{P}$= 81.4 CPIs/week.

The average prevalence and weekly incidence of ESBL-EC in the i-Bird study, considered as steady-state values, are (from Table 1):$c_{EC}^{*}$= 11.51% and $i_{EC}^{*}$ =1.96%/week

Hence, the duration of ESBL-EC colonization may be estimated as:

D_EC_ = $c_{EC}^{*}$/$i_{EC}^{*}$ = 5.9 weeks

⇒ $\gamma_{EC}$ = 1/D_EC_ = 0.17 (weeks)^–1^

Based on our analysis of incident cases and potential infectors, the proportion of cases of incident ESBL-EC colonization with a potential infector identified at a distance ≤ 2 is:

$\tau_{EC}$= 51%

Hence the weekly rate of ESBL-EC acquisition from the endogenous route is estimated at:

${}_{EC}= i_{EC}^{*}\times\left( 1-\tau_{EC} \right)=1.96\%\times\left( 1-0.51 \right)=0.96\%$/week

And the per-contact probability of ESBL-EC transmission may be computed as:

$p_{EC}=\frac{1}{c_{P}}\times\left( \frac{\gamma_{EC}}{s_{EC}^{*}}-\frac{{}_{EC}}{c_{EC}^{*}} \right)=\frac{1}{81.4}\times\left( \frac{0.17}{1-11.51\%}-\frac{0.96\%}{11.51\%} \right)=0.134\%$/contact

***Numerical application for ESBL-KP***

Based on the i-Bird CPI data, the baseline per-patient average weekly number of CPIs at a distance ≤ 2 is $c_{P}$=81.4 CPIs/week.

The average prevalence and weekly incidence of ESBL-KP in the i-Bird study, considered as steady-state values, are (from Table 1):$c_{KP}^{*}$= 3.73% and $i_{KP}^{*}$ =1.15%/week

Hence, the duration of ESBL-KP colonization may be estimated as:

*D_KP_* = $c_{KP}^{*}$ /$i_{KP}^{*}$ = 3.2 weeks

⇒ $\gamma_{KP}$ = 1/ *D_KP_* = 0.31 (weeks)^–1^

Based on our analysis of incident cases and potential infectors, the proportion of cases of incident ESBL-KP colonization with a potential infector identified at a distance ≤ 2 is:

$\tau_{KP}$= 75%

Hence the weekly rate of ESBL-KP acquisition from the endogenous route or the environment is estimated at:

${}_{KP}= i_{KP}^{*}\times\left( 1-\tau_{KP} \right)=1.15\%\times\left( 1-0.75 \right)=0.29\%$/week

And the per-contact probability of ESBL-KP transmission may be computed as:

$p_{KP}=\frac{1}{c_{P}}\times\left( \frac{\gamma_{KP}}{s_{KP}^{*}}-\frac{{}_{KP}}{c_{KP}^{*}} \right)=\frac{1}{81.4}\times\left( \frac{0.31}{1-3.73\%}-\frac{0.29\%}{3.73\%} \right)=0.299\%$/contact

**Model Output: Reduction in the cumulative incidence**

For each scenario *s* and each bacterium, the model was run for 17 weeks and the cumulative incidence of acquisitions was calculated ($\sum_{1}^{17} Inc(w))$. Then the reduction of cumulative incidence for a given scenario compared with a scenario with no intervention was calculated as follows:

$$RCI= \frac{I_{cb}- I_{cs}}{I_{cb}}$$

Where $I_{cb}$ represents the 4-month cumulative incidence under the baseline scenario and $I_{cs}$ the 4-month cumulative incidence under the intervention scenarios.
