# Supplementary figures and images for: Close proximity interactions support transmission of ESBL-K. pneumoniae but not ESBL-E. coli in healthcare settings
Source: PLoS Comput Biol. 2019 May 30;15(5):e1006496. doi: 10.1371/journal.pcbi.1006496 (PMC6542504; doi:10.1371/journal.pcbi.1006496)

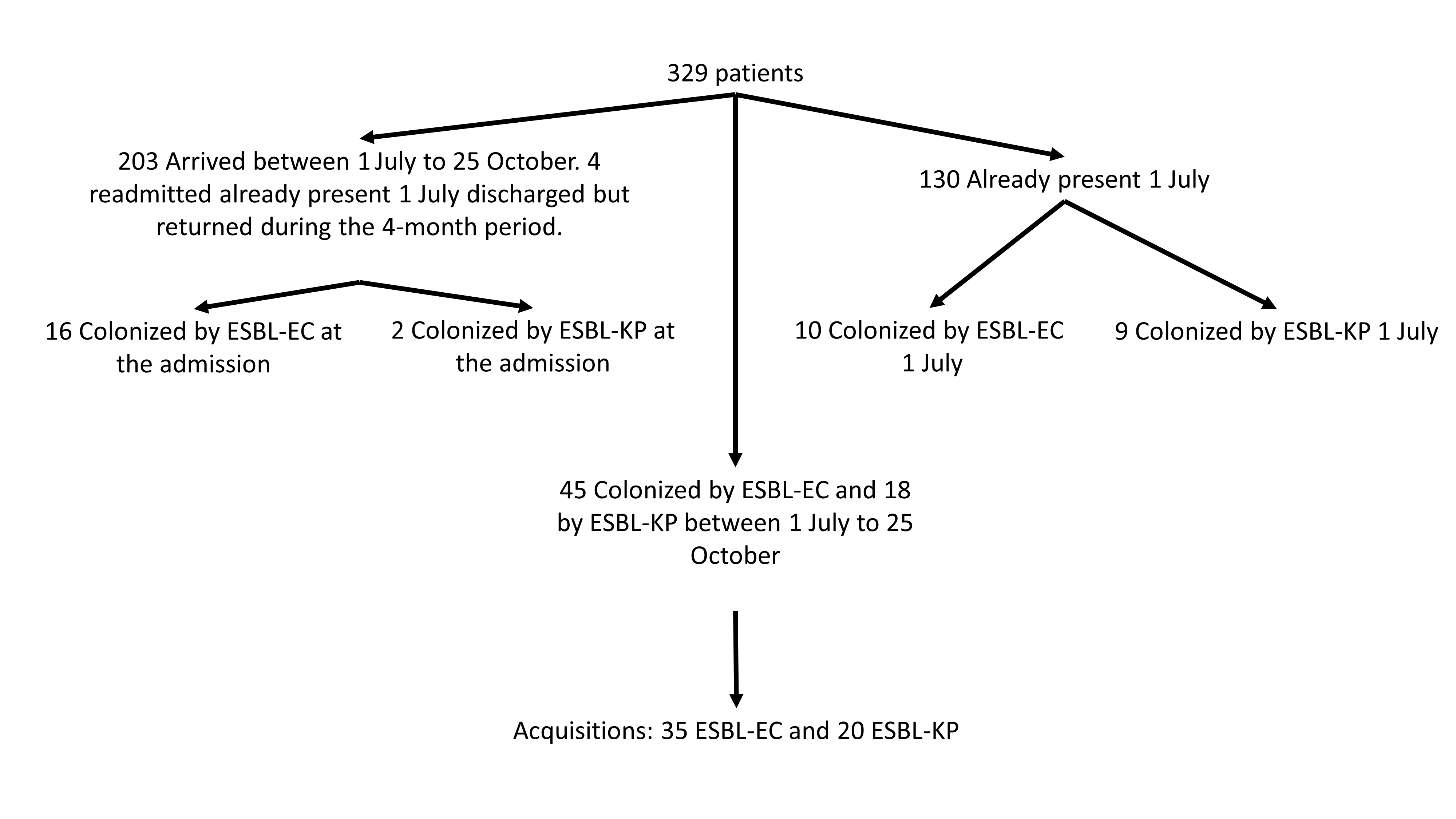

Supplement: S1 Fig — (TIF) [file pcbi.1006496.s007.TIF]

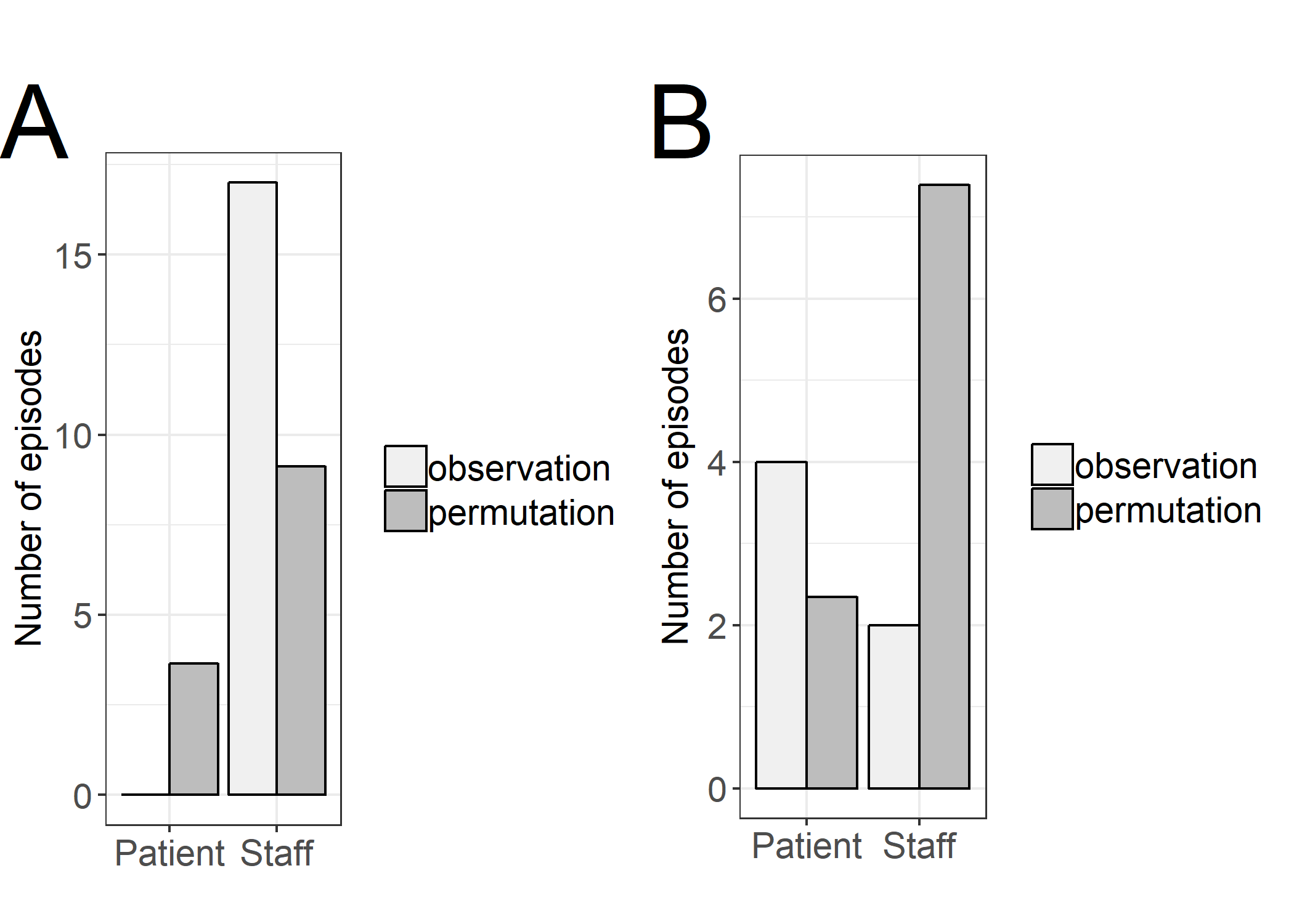

Supplement: S2 Fig — Here, only incident-colonization episodes with a distance-2 to their potential infector are considered. The portions of these episodes in which there is a majority of patients and hospital staff are depicted for (A) ESBL-EC and (B) ESBL-KP. These portions are compared between observed (light grey) and randomly permutated data (dark grey). (TIFF) [file pcbi.1006496.s008.tiff]

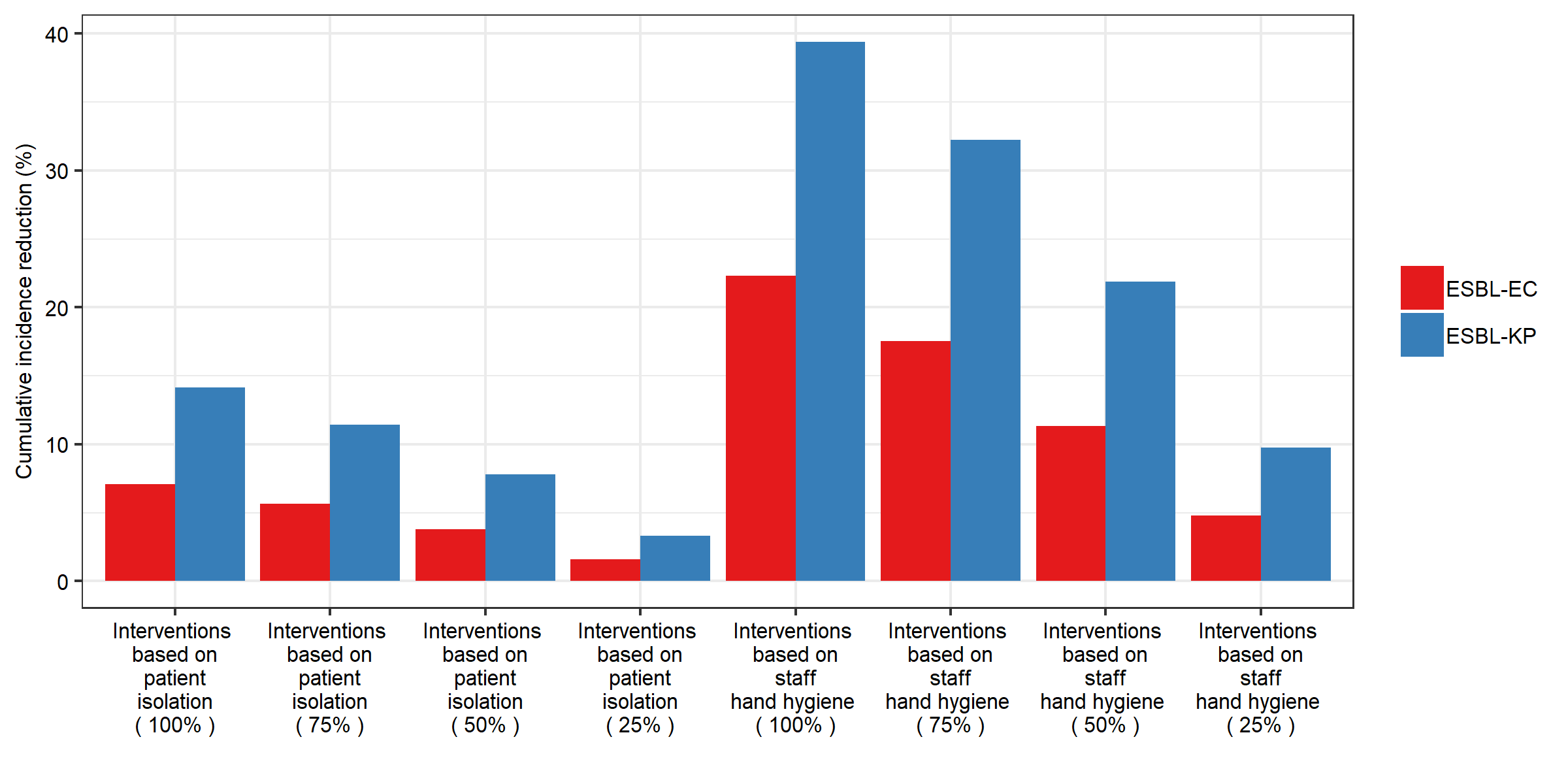

Supplement: S3 Fig — Percentage on the y-axis corresponds to reduction of the cumulative incidence compared to the baseline (scenario with no control measure). In red, percentage of cumulative incidence reduction of ESBL-EC and blue ESBL-KP. Intervention based on patient isolation correspond to a removal of 100%, 75%, 50% and 25% of patient-patient CPIs. Intervention based on staff hand hygiene correspond to a removal of 100%, 75%, 50% and 25% of patient-staff CPIs. (TIFF) [file pcbi.1006496.s009.tiff]

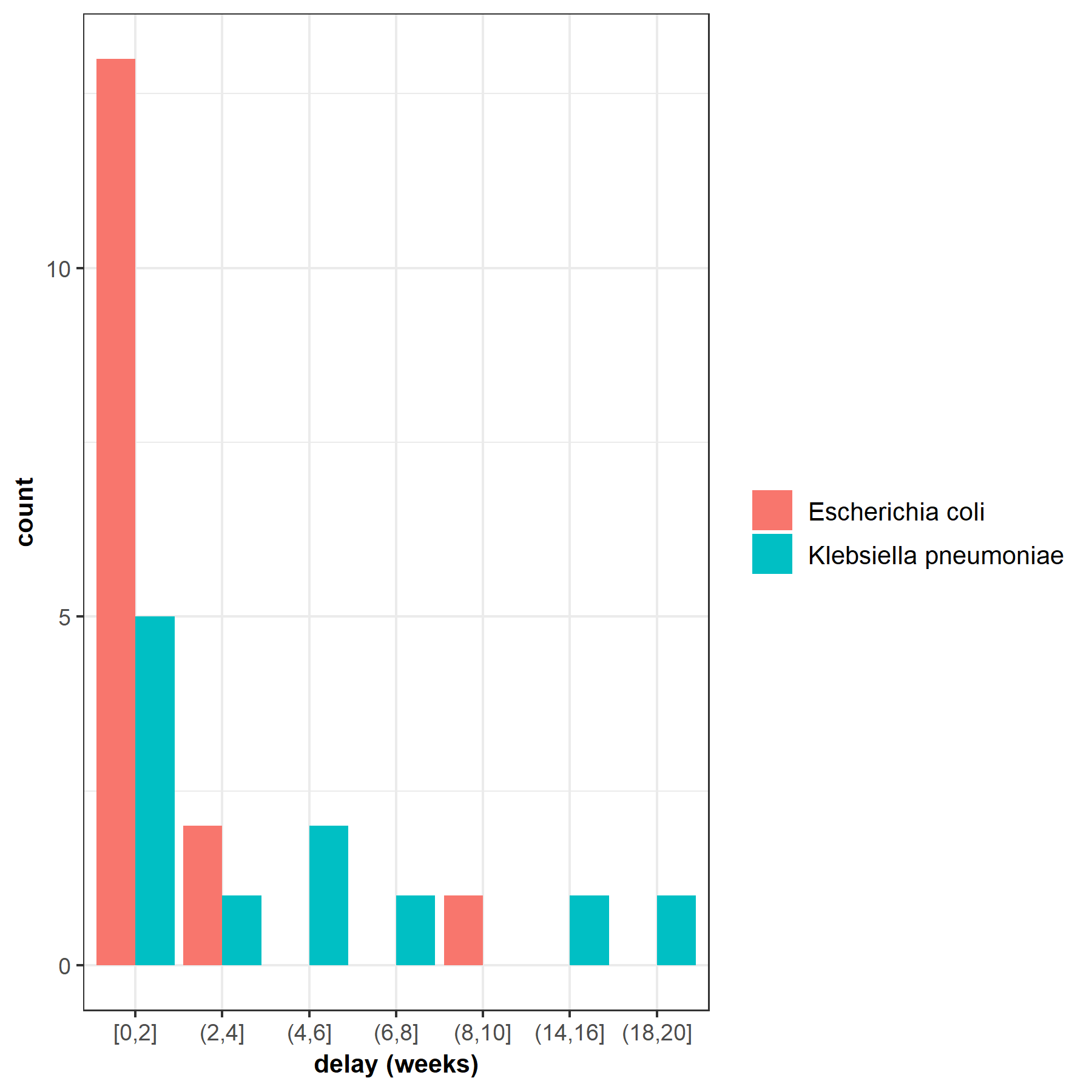

Supplement: S4 Fig — The distribution of the delay between the last day of antibiotic use and the colonization event day is depicted for both ESBL-EC and ESBL-KP. Delays were shorter for ESBL-EC than for ESBL-KP, leading to fewer swabs in the interval. (TIFF) [file pcbi.1006496.s010.tiff]

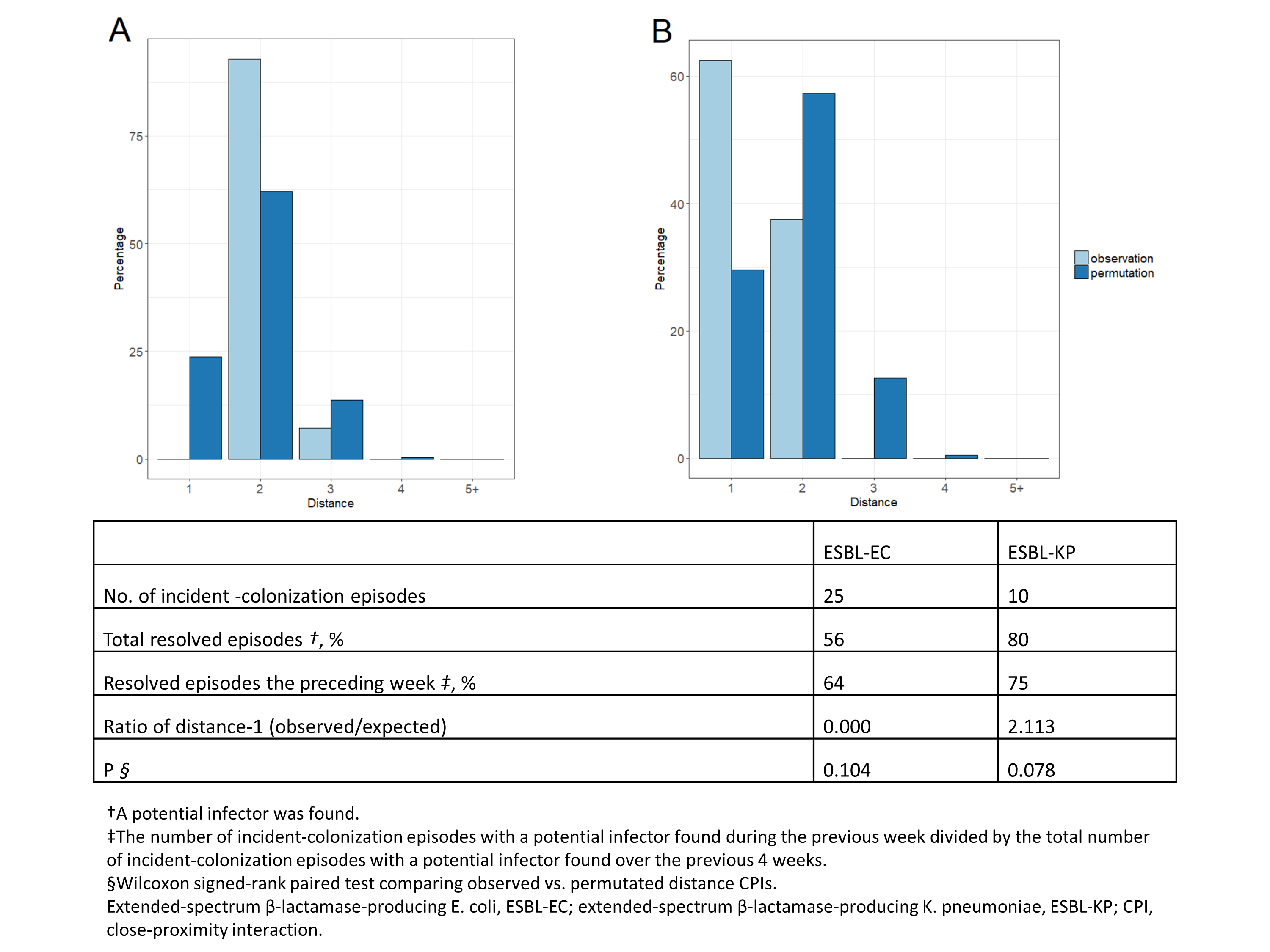

Supplement: S5 Fig — Incident-colonization episodes were defined as two negatives swabs followed by one positive swab for a given ESBL-EC or ESBL-KP isolate. Comparison between observed data (light blue) and random permutated data (dark blue). For each incident-colonization case, potential infectors were selected as the closest in the CPI-network of all candidates sharing the most similar isolate as the case in the preceding 4 weeks. (A) ESBL-EC distribution. (B) ESBL-KP distribution. Here distance is the number of edge between two individuals in the network. Because of the few episodes resolved (14/25 and 8/10 for ESBL-EC and ESBL-KP respectively), expected distances were computed as the average of distances obtained from 500 instead of 200 simulations using randomly permutated carriage data. (TIF) [file pcbi.1006496.s011.tif]

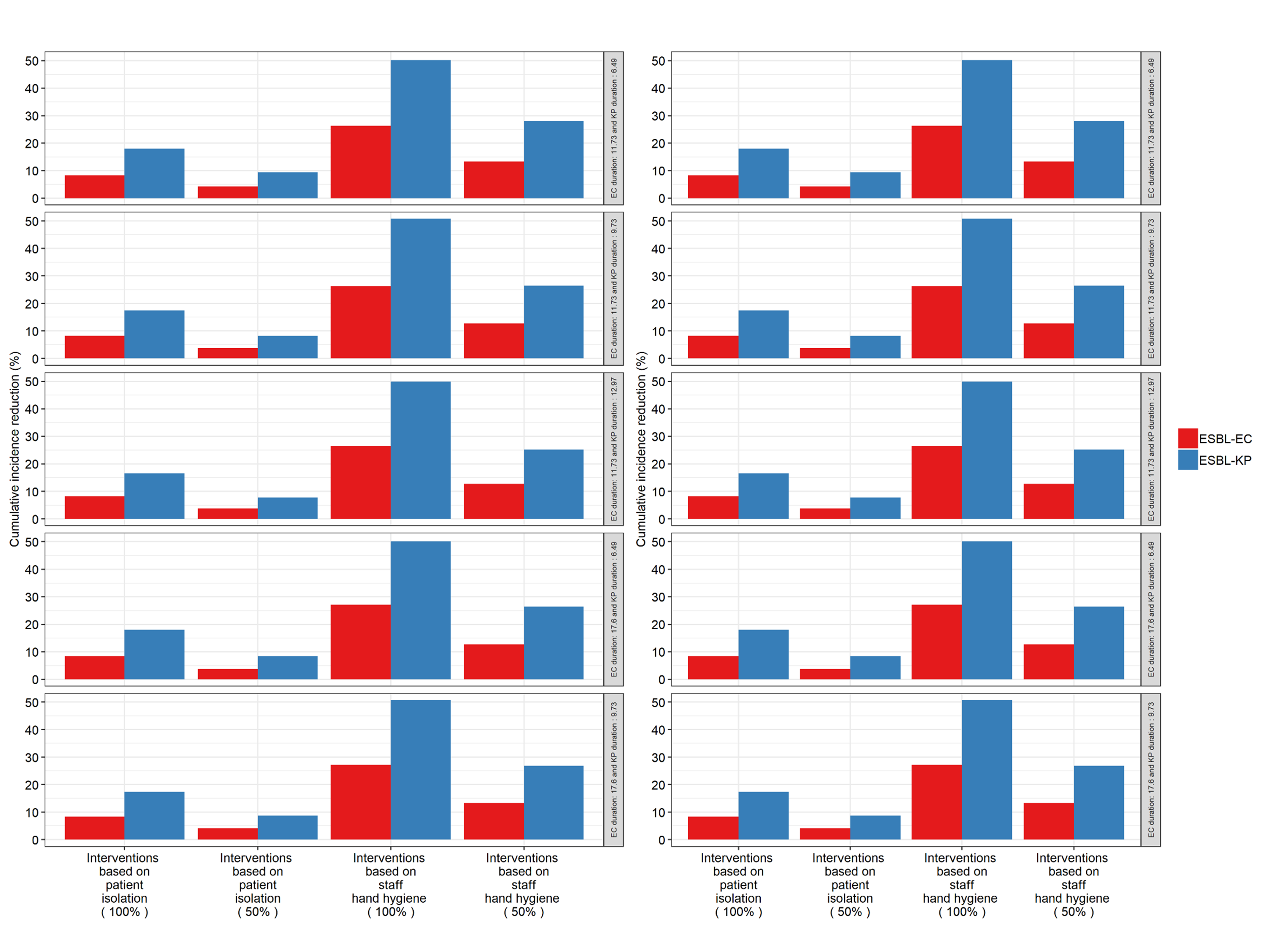

Supplement: S6 Fig — Different durations of carriage were used: 11.73, 17.60 and 23.47 weeks for ESBL-EC and 6.49, 9.73 and 12.97 weeks for ESBL-KP. The percentage on the y-axis corresponds to the reduction of the cumulative incidence compared to the baseline scenario (with no control measure), for ESBL-EC (in red) or ESBL-KP (in blue). Interventions based on patient isolation correspond to a removal of 100% or 50% of patient-patient CPIs. Intervention based on staff hand hygiene correspond to a removal of 100% or 50% of patient-staff CPIs. The assumed duration of carriage for ESBL-EC and ESBL-KP is mentioned in the grey rectangle at the right of each graph. No matter the duration of carriage, reduction of cumulative incidence is more important for ESBL-KP than for ESBL-EC and more efficient when the intervention is based on staff hand hygiene. (TIF) [file pcbi.1006496.s012.tif]

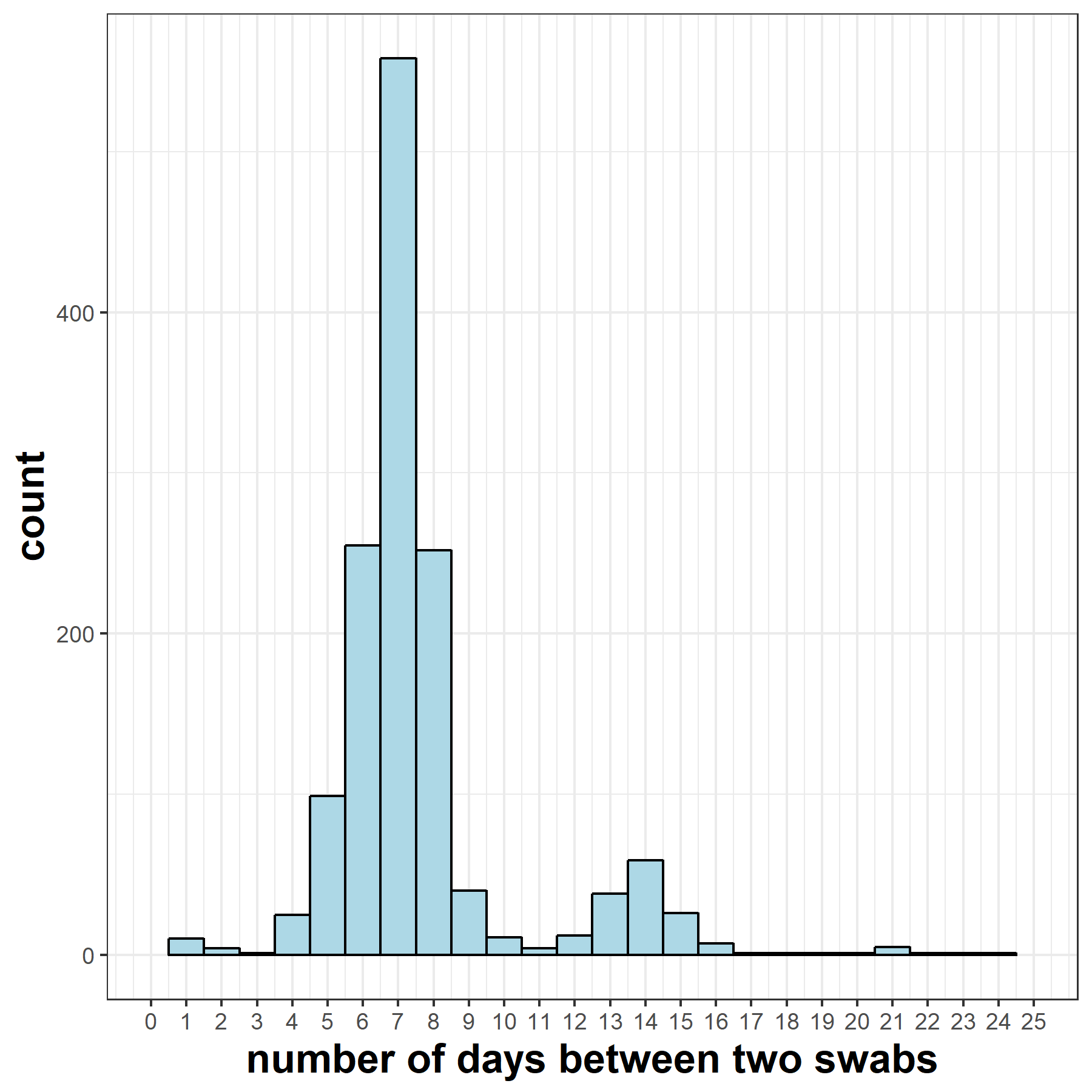

Supplement: S7 Fig — The time interval between two swabs was mostly at 7 days. (TIFF) [file pcbi.1006496.s013.tiff]

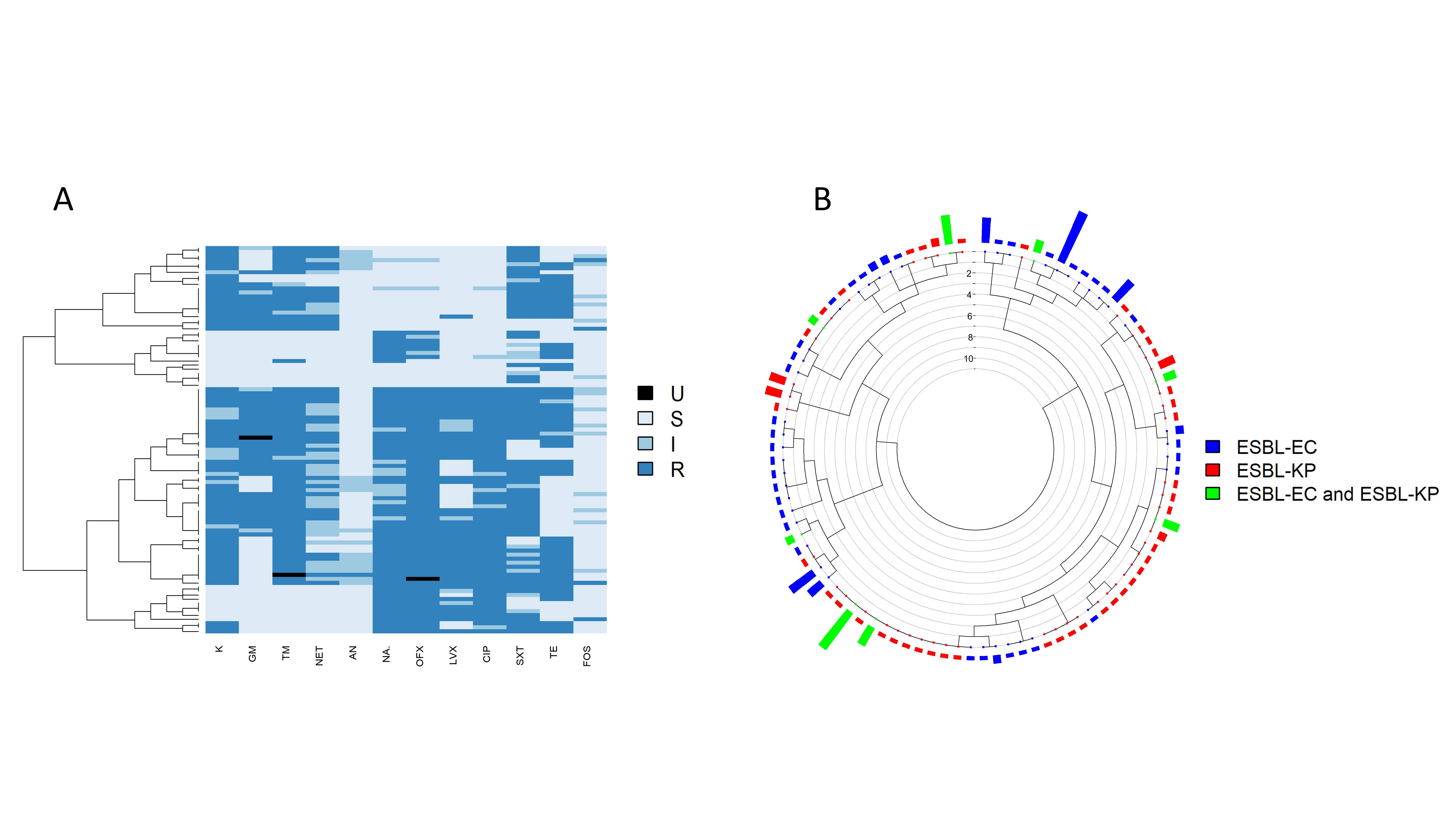

Supplement: S8 Fig — (A), Each row represents an isolate identified during the study. Each column represents the phenotype sequence in terms of antibiotic resistance level to each of the 12 tested antibiotics. R, resistant (dark blue). I, intermediate (blue). S, susceptible (light blue) and U unknown (black). Tested antibiotics were penicillins (aminoglycosides (kanamycin (K), gentamicin (GM), tobramycin (TM), netilmicin (NET), amikacin (AN)), fluoroquinolones (nalidixic acid (NA), ofloxacin (OFX), levofloxacin (LVX), ciprofloxacin (CIP)), co-trimoxazole (SXT), tetracyclines (TE) and fosfomycin (FOS). The dendrogram was built from the distances between two phenotype profiles for the 12 antibiotics. (B) The same data is represented with characterization of the species. Blue: ESBL-EC, red: ESBL-KP and green: resistance sequences found in both species. Rectangle heights correspond to the number of individuals each profile was observed in. (TIF) [file pcbi.1006496.s014.tif]

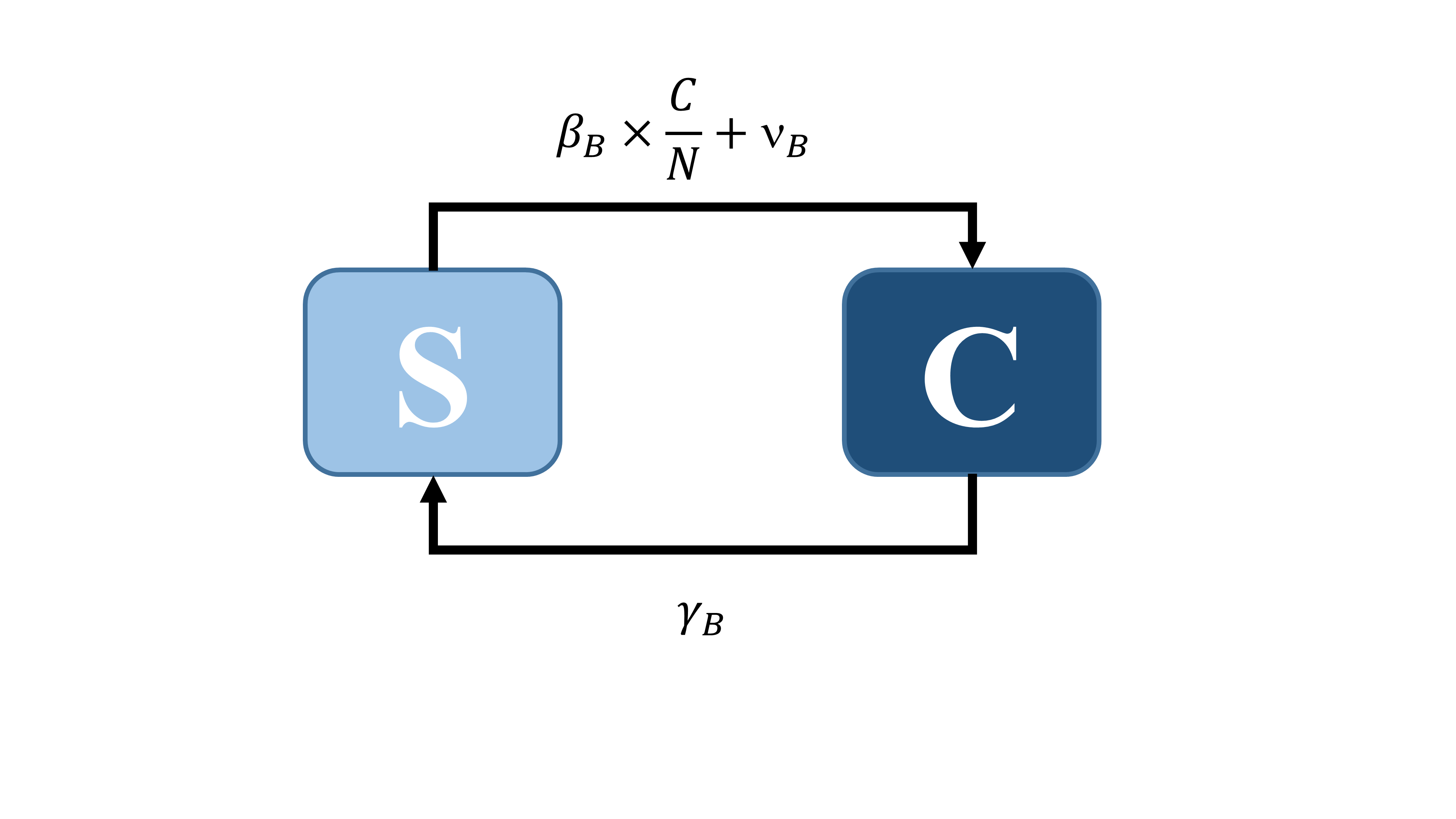

Supplement: S9 Fig — S and C are the susceptible and colonized compartments. βB is the weekly effective contact rate, N is the total number of patients within the LTCF, νB is the weekly colonization-acquisition rate via the endogenous route or the environment and γB is the decolonization rate of bacteria B. (TIF) [file pcbi.1006496.s015.tif]
