# Supplementary material for: Close proximity interactions support transmission of ESBL-K. pneumoniae but not ESBL-E. coli in healthcare settings
Source: PLoS Comput Biol. 2019 May 30;15(5):e1006496. doi: 10.1371/journal.pcbi.1006496 (PMC6542504; doi:10.1371/journal.pcbi.1006496)
Supplement: S1 Table — Generalized linear model performed on 6 isolates involved in the acquisition of more than one patient. P-values were computed with a likelihood ratio test. “Other” gathers administrative, logistic and animation staff. (DOCX) [file pcbi.1006496.s016.docx]

|  | | OR | CI 95% | p-value |
| --- | --- | --- | --- | --- |
| ESBL-EC isolate 1  hospital worker profession (ref: auxiliary nurses) | ASH | 0.310 | (0.067-1.424) | 9.50E-04 |
|  | Hospital porter | 1.162 | (0.204-6.625) |  |
|  | Physician | 0.000 | (0-∞) |  |
|  | Nurse | 2.324 | (1.222-4.421)* |  |
|  | Other | 0.303 | (0.086-1.072). |  |
|  | Reeducation staff | 0.979 | (0.393-2.435) |  |
| ESBL-EC isolate 2  hospital worker profession (ref: auxiliary nurses) | ASH | 1.400 | (0.497-3.945) | 3.56E-03 |
|  | Hospital porter | 10.000 | (1.131-88.424)* |  |
|  | Physician | 2.000 | (0.387-10.348) |  |
|  | Nurse | 2.000 | (1.058-3.781)* |  |
|  | Other | 0.364 | (0.118-1.125). |  |
|  | Reeducation staff | 0.571 | (0.214-1.525) |  |
| ESBL-EC isolate 3  hospital worker profession (ref: auxiliary nurses) | ASH | 0.892 | (0.186-4.270) | 1.96E-02 |
|  | Hospital porter | 6.688 | (1.241-36.039)* |  |
|  | Physician | 3.344 | (0.566-19.766) |  |
|  | Nurse | 2.128 | (0.957-4.729). |  |
|  | Other | 0.268 | (0.034-2.112) |  |
|  | Reeducation staff | 2.816 | (1.058-7.495)* |  |
| ESBL-KP isolate 1  hospital worker profession (ref: auxiliary nurses) | ASH | 0.674 | (0.223-2.034) | 1.34E-02 |
|  | Hospital porter | 3.234 | (0.570-18.351) |  |
|  | Physician | 0.809 | (0.142-4.588) |  |
|  | Nurse | 1.141 | (0.604-2.157) |  |
|  | Other | 0.135 | (0.030-0.597)** |  |
|  | Reeducation staff | 0.566 | (0.222-1.441) |  |
| ESBL-KP isolate 2  hospital worker profession (ref: auxiliary nurses) | ASH | 1.220 | (0.420-3.542) | 4.20E-04 |
|  | Hospital porter | 4.474 | (0.785-25.487). |  |
|  | Physician | 2.237 | (0.432-11.594) |  |
|  | Nurse | 1.270 | (0.657-2.452) |  |
|  | Other | 0.000 | (0-∞) |  |
|  | Reeducation staff | 0.783 | (0.305-2.008) |  |
| ESBL-KP isolate 3  hospital worker profession (ref: auxiliary nurses) | ASH | 1.844 | (0.662-5.137) | 1.03E-03 |
|  | Hospital porter | 10.375 | (1.173-91.777)* |  |
|  | Physician | 0.415 | (0.047-3.671) |  |
|  | Nurse | 1.686 | (0.889-3.199) |  |
|  | Other | 0.271 | (0.077-0.955)* |  |
|  | Reeducation staff | 2.594 | (1.111-6.054)* |  |
| ***Note: Observed level of the Wald test for each parameter: * <0.05, ** <0.01, *** <0.001. OR: odds ratio.*** | | | | |
